# *Sonic hedgehog *expressing and responding cells generate neuronal diversity in the medial amygdala

**DOI:** 10.1186/1749-8104-5-14

**Published:** 2010-05-27

**Authors:** Rosalind SE Carney, Jean-Marie Mangin, Lindsay Hayes, Kevin Mansfield, Vitor H Sousa, Gord Fishell, Robert P Machold, Sohyun Ahn, Vittorio Gallo, Joshua G Corbin

**Affiliations:** 1 Center for Neuroscience Research, Children's Research Institute, Children's National Medical Center, Washington, DC 20010, USA; 2 Unit on Developmental Neurogenetics, Program in Genomics of Differentiation, Eunice Kennedy Shriver National Institute of Child Health and Human Development, Bethesda, MD 20892, USA; 3 Neuroscience Program and Department of Cell Biology, Smilow Research Center, New York University School of Medicine, New York, NY 10016, USA; 4 Present address: Molecular Neurobiology Laboratory (MNL-O), The Salk Institute, 10010 North Torrey Pines Road, La Jolla, CA 92037, USA

## Abstract

**Background:**

The mammalian amygdala is composed of two primary functional subdivisions, classified according to whether the major output projection of each nucleus is excitatory or inhibitory. The posterior dorsal and ventral subdivisions of the medial amygdala, which primarily contain inhibitory output neurons, modulate specific aspects of innate socio-sexual and aggressive behaviors. However, the development of the neuronal diversity of this complex and important structure remains to be fully elucidated.

**Results:**

Using a combination of genetic fate-mapping and loss-of-function analyses, we examined the contribution and function of *Sonic hedgehog *(*Shh*)-expressing and *Shh*-responsive (*Nkx2-1*^+ ^and *Gli1*^+^) neurons in the medial amygdala. Specifically, we found that *Shh- *and *Nkx2-1-*lineage cells contribute differentially to the dorsal and ventral subdivisions of the postnatal medial amygdala. These *Shh*- and *Nkx2-1*-lineage neurons express overlapping and non-overlapping inhibitory neuronal markers, such as Calbindin, FoxP2, nNOS and Somatostatin, revealing diverse fate contributions in discrete medial amygdala nuclear subdivisions. Electrophysiological analysis of the *Shh*-derived neurons additionally reveals an important functional diversity within this lineage in the medial amygdala. Moreover, inducible *Gli1^CreER(T2) ^*temporal fate mapping shows that early-generated progenitors that respond to *Shh *signaling also contribute to medial amygdala neuronal diversity. Lastly, analysis of *Nkx2-1 *mutant mice demonstrates a genetic requirement for *Nkx2-1 *in inhibitory neuronal specification in the medial amygdala distinct from the requirement for *Nkx2-1 *in cerebral cortical development.

**Conclusions:**

Taken together, these data reveal a differential contribution of *Shh-*expressing and *Shh*-responding cells to medial amygdala neuronal diversity as well as the function of *Nkx2-1 *in the development of this important limbic system structure.

## Background

The mammalian amygdala is an aggregation of 11 to 15 nuclei, which as components of the limbic system mediate distinct aspects of emotional and behavioral processing as well as socio-sexual behaviors (reviewed in [1]). The amygdala is considered in terms of two functional subdivisions, classified according to whether the major output projection of each nucleus is excitatory or inhibitory. Based on this classification, the nuclei of the basolateral complex and cortical amygdalar nuclei, which have an excitatory output, have been hypothesized to be of a pallial origin, whereas the cortical and medial nuclei are broadly considered similar to the striatum as their output projections are primarily inhibitory. Several studies have revealed that, during embryogenesis, the emerging amygdala is generated from several forebrain embryonic domains, including the pallium, pallial-subpallial boundary, medial ganglionic eminence (MGE), preoptic area (POA) and perhaps the diencephalon [2-10].

The medial amygdala (MeA) subnuclei process pheromonal information, and regulate neuroendocrine function and socio-sexual behaviors. Anatomically, the posterior portion of the MeA is divided into dorsal (medial posterodorsal nucleus (MePD)) and ventral (medial posteroventral nucleus (MePV)) subdivisions, which via their projections to distinct hypothalamic nuclei regulate reproductive and defensive behaviors, respectively (reviewed in [1,11]). Although not well-characterized, recent studies have revealed insight into the development of the MeA. In terms of circuit formation, the anatomical segregation of efferent projections that regulate reproductive or defensive behaviors is differentially marked by the LIM-containing homeodomain genes *Lhx6 *and *Lhx9 *[12,13]. In the embryonic subpallium, *Lhx6 *is expressed in tangentially migrating cortical interneurons [14-16], and is a direct transcriptional target of *Nkx2-1*, which is expressed in the embryonic MGE and POA [17,18]. In addition, our recent work has revealed that the embryonic telencephalic POA is a major novel source of MeA neurons [3]. Moreover, *Nkx2-1*-lineage cells have been shown to contribute to the anterior MeA, although their neurochemical fate was not determined [5]. Despite this developmental information, the lineages that contribute to the vast inhibitory neuronal diversity in the MeA remain largely unknown.

Based on the expression patterns of the morphogen Sonic hedgehog (*Shh*) as well as the *Shh*-responsive genes *Nkx2-1 *and *Gli1 *in the embryonic MGE and POA [19,20] and, at later developmental stages (by embryonic day 14.5 (E14.5)), expression of *Shh *in the MePV [2], we hypothesized that progenitor populations marked by these genes contribute to inhibitory neuronal diversity in the postnatal MeA. In this study, we find that *Shh-*, *Nkx2-1- *and *Gli1*-lineage cells generate inhibitory neuronal diversity in the MePD and MePV in a largely complementary manner. We further reveal a differential functional requirement for *Nkx2-1 *in the development of the MePD compared to the MePV, and find that *Shh*-lineage cells generate three functionally distinct classes of neurons in the posterior MeA as defined by electrophysiological criteria. Thus, these data provide novel insights into the lineage and genetic mechanisms that generate the amygdala nuclei that mediate socio-sexual behaviors.

## Results

### Embryonic expression of *Nkx2-1*, *Shh *and *Gli1 *and recombined cells

Progenitor pools in the embryonic telencephalon are marked by spatio-temporal diversity in gene expression patterns. Expression of these genes is typically down-regulated as cells become post-mitotic. To permanently label these transiently marked populations, we used transgenic mouse lines that express Cre recombinase under transcriptional regulation of *Shh*, *Nkx2-1 *and *Gli1 *[5,21,22]. The developmental expression of these genes has been well-characterized in previous studies. At E11.5 and E13.5, *Nkx2-1 *is expressed throughout the MGE progenitor domains (pMGE 1-5) and the progenitor domains of the dorsal (pPOA1) and ventral (pPOA2) subdivisions of the embryonic POA [19]. In addition, previous studies [5] revealed that *Nkx2-1*-derived cells, using the same *Nkx2-1*-*Cre *mouse line as in this study, migrate toward the developing amygdala. At E11.5, *Shh *is expressed in the MGE mantle zone and in the progenitor domains of the POA (pPOA1 and pPOA2) and in a small region of the septum [19,23]. *Gli1 *is a transcriptional target of *Shh* and its expression, therefore, is observed near *Shh*-expressing domains [24-26]. Consequently, at early developmental time points *Gli1 *mRNA expression is observed in the ventral POA (pPOA2) and the sulcus between the lateral ganglionic eminence (LGE) and the MGE (pLGE4 and pMGE1) [20,27].

**Figure 1:**
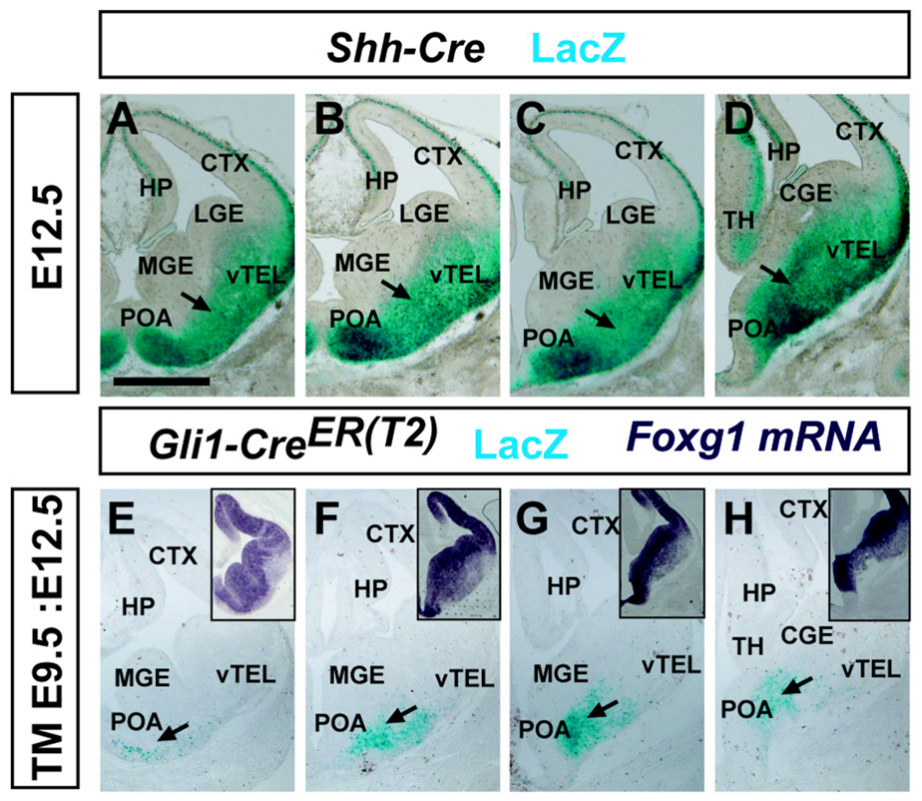
**Short-term recombination in *Shh*^*Cre *^and inducible *Gli1*^*CreER(T2) *^lines**. **(A-D) **Rostro-caudal sequence from coronal sections of E12.5 *Shh*^*Cre*^*; Tau*^*mGFP *^brains (*n *= 2), where LacZ staining reveals recombined cells in the region of the MGE, POA and ventral telencephalon (vTEL) (arrows) and, to a lesser extent, in the thalamus (TH), cortex (CTX) and hippocampus (HP). **(E-H) **A single dose of tamoxifen (TM) administered at E9.5 labeled cells at approximately E10.5 in *Gli1*^*CreER(T2)*^*; Tau*^*mGFP *^brains (*n *= 2). The inducible *Gli1*^*CreER(T2) *^line was used to identify *Shh*-responding cells during a temporally restricted time window. A rostro-caudal sequence shows LacZ^+ ^cells in the POA and vTEL (arrows). Adjacent sections, processed for *Foxg1 *mRNA expression (insets), show that the recombined cells are in telencephalic domains of the forebrain. Abbreviation: LGE, lateral ganglionic eminence. Scale bar: 150 μm.

As *Tau*^*mGFP *^mice were generated with a nuclear localization signal for LacZ [28], we used X-gal staining to visualize recombined cells. Our short-term recombination analyses at E12.5 revealed waves of putative migrating cells emanating from the ventral telencephalon to the prospective developing amygdaloid region. In the *Shh*^*Cre*^*; Tau*^*mGFP *^line we observed intense X-gal staining in the post-mitotic regions of the MGE, POA and ventral telencephalon (Figure 1A-D). *Shh*-lineage cells were also observed to a lesser extent in the developing thalamus, cerebral cortex and hippocampus (Figure 1A-D). This result is also consistent with a recent study using the ROSA26 LacZ reporter mouse that showed *Shh*-lineage cells also in domains of the POA and MGE [29]. As MeA neurons are generated from as early as E10 [3,4], we administered a single dose of tamoxifen (TM) at E9.5 (TM E9.5) in pregnant dams from *Gli1*^*CreER(T2)*^; *Tau*^*mGFP *^crosses to label *Shh*-responder cells (*Gli1*^+^) at approximately E10.5. When analyzed at E12.5, X-gal staining was observed in a number of cells in the post-mitotic region of the MGE and POA (Figure 1E-H), similar to that observed for the *Shh*^*Cre *^line. Furthermore, *Foxg1 *mRNA expression (Figure 1E-H, insets) on adjacent sections showed that the basally located short-term recombined cells in the *Shh*^*Cre *^and *Gli1*^*CreER(T2) *^lines were located in the telencephalon as opposed to the diencephalon.

In summary, based on our analyses and previous studies, *Shh*, *Nkx2-1 *and *Gli1 *are spatially and temporally present in progenitor domains known to contribute to the MeA. Therefore, we next focused our analyses on the postnatal characterization of these genetically labeled cell populations.

### *Nkx2-1*- and *Shh*-lineage cells preferentially accumulate in different subdivisions of the adult posterior MeA

**Figure 2:**
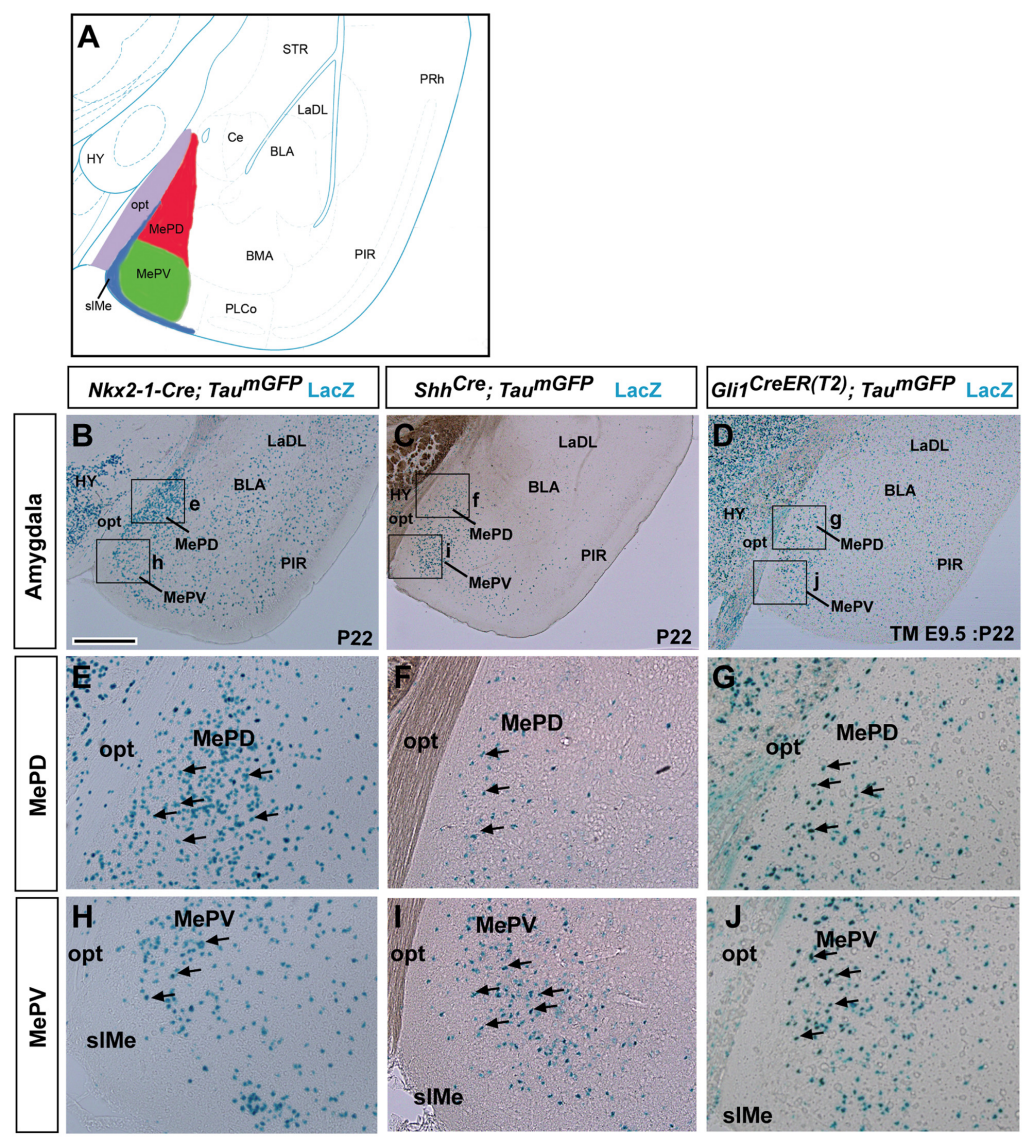
***Nkx2-1*- and *Shh*-lineage cells preferentially fate-map to complementary subdivisions of the posterior medial amygdala**. **(A) **Schema of a caudal coronal section of an adult mouse brain showing the nuclei of the amygdala. The medial posterodorsal nucleus (MePD; red) is located lateral to the optic tract (opt; purple), and the medial posteroventral nucleus (MePV; green) is located ventrally and is bordered by the superficial layer of the medial nucleus (slMe; blue), which is a cell sparse fiber tract. **(B,E,H) **LacZ staining in postnatal day 22 (P22) *Nkx2-1-Cre; Tau*^*mGFP *^brains (*n *= 2) revealed that most recombined cells are found in the MePD (E, arrows), with fewer cells in the MePV (H, arrows). **(C,F,I) ***Shh*-producing cells revealed the opposite whereby more LacZ^+ ^cells were observed in the MePV (I, arrows) of P22 *Shh*^*Cre*^*; Tau*^*mGFP *^brains (*n *= 2) than the MePD (F, arrows). **(D,G,J) **A single dose of tamoxifen administered at E9.5 (TM E9.5: P22; tamoxifen at E9 and survival to P22) resulted in numerous LacZ^+ ^cells in *Gli1*^*CreER(T2)*^*; Tau*^*mGFP *^brains (*n *= 2). In this case, more recombined cells were observed in the MePD than MePV (G,J, arrows) of the posterior MeA. Abbreviations: BLA, basolateral amygdala nucleus; BMA, basomedial amygdala nucleus; Ce, central nucleus; HY, hypothalamus; LaDL, dorsolateral subdivision of the lateral amygdala nucleus; PIR, piriform cortex; PLCo, posterolateral cortical amygdala nucleus; PRh, perirhinal cortex; STR, striatum. Scale bar: 600 μm (B-D); 200 μm (E-J).

To determine whether *Shh*-, *Nkx2-1*- and *Gli1-*derived cells contribute to the dorsal and ventral subdivisions of the posterior MeA (Figure 2A), we analyzed their distribution patterns at postnatal day 22 (P22). In *Nkx2-1-Cre; Tau*^*mGFP *^brains LacZ^+ ^cells were predominantly located in the MePD (Figure 2B, E, H). In addition, *Shh*-lineage cells contributed substantially to the posterior MeA (Figure 2C, F, I). Interestingly, this distribution of *Shh*-lineage cells appeared largely complementary to that of the *Nkx2-1-*lineage population. In contrast to these primarily complementary patterns of recombination, *Shh*-responding cells (*Gli1*-derived) labeled at approximately E10.5 (TM E9.5) were distributed between both the MePD and MePV (Figure 2D, G, J).

Taken together, these results show that the dorsal (MePD) and ventral (MePV) subdivisions of the adult posterior MeA can be preferentially populated from *Nkx2-1*- and *Shh*-lineages, respectively. *Nkx2-1*-lineage cells are predominantly observed in the MePD whereas *Shh*-lineage cells are mainly observed in the MePV. In addition, we show that *Gli1*-derived cells contribute to both subdivisions of the posterior MeA.

### Characterization of inhibitory neuronal cell types in the posterior MeA

Neuronal cell diversity in the MeA has not been thoroughly explored. Therefore, we first wanted to characterize the molecular/neurochemical profiles of specific cell types in both the MePD and MePV. We reasoned that this analysis was a necessary prerequisite for subsequent analysis of the fate of *Nkx2-1*-, *Shh*- and *Gli1*-derived populations. To this end, we immunostained P22 wild-type mice with a battery of antibodies against proteins whose expression patterns are well-characterized in other forebrain structures but have not been directly compared to or between the subdivisions of the posterior MeA. As the MeA is largely composed of GABAergic neurons (reviewed in [1]), expression of Tbr1, which is a marker of excitatory neurons [30,31], was only observed in the ventral-most portion of the MePV, adjacent to the superficial layer of the MeA (Additional file 1A, B). Conversely both the MePD and MePV were highly immunoreactive for the GABA synthesizing enzymes GAD65/67 (Additional file 1C, D). Surprisingly, we found that expression of Parvalbumin and Neuropeptide Y, which are markers of inhibitory neuronal subtypes in the cerebral cortex and striatum, were nearly absent from the posterior MeA (Additional file 1E-H).

**Figure 3:**
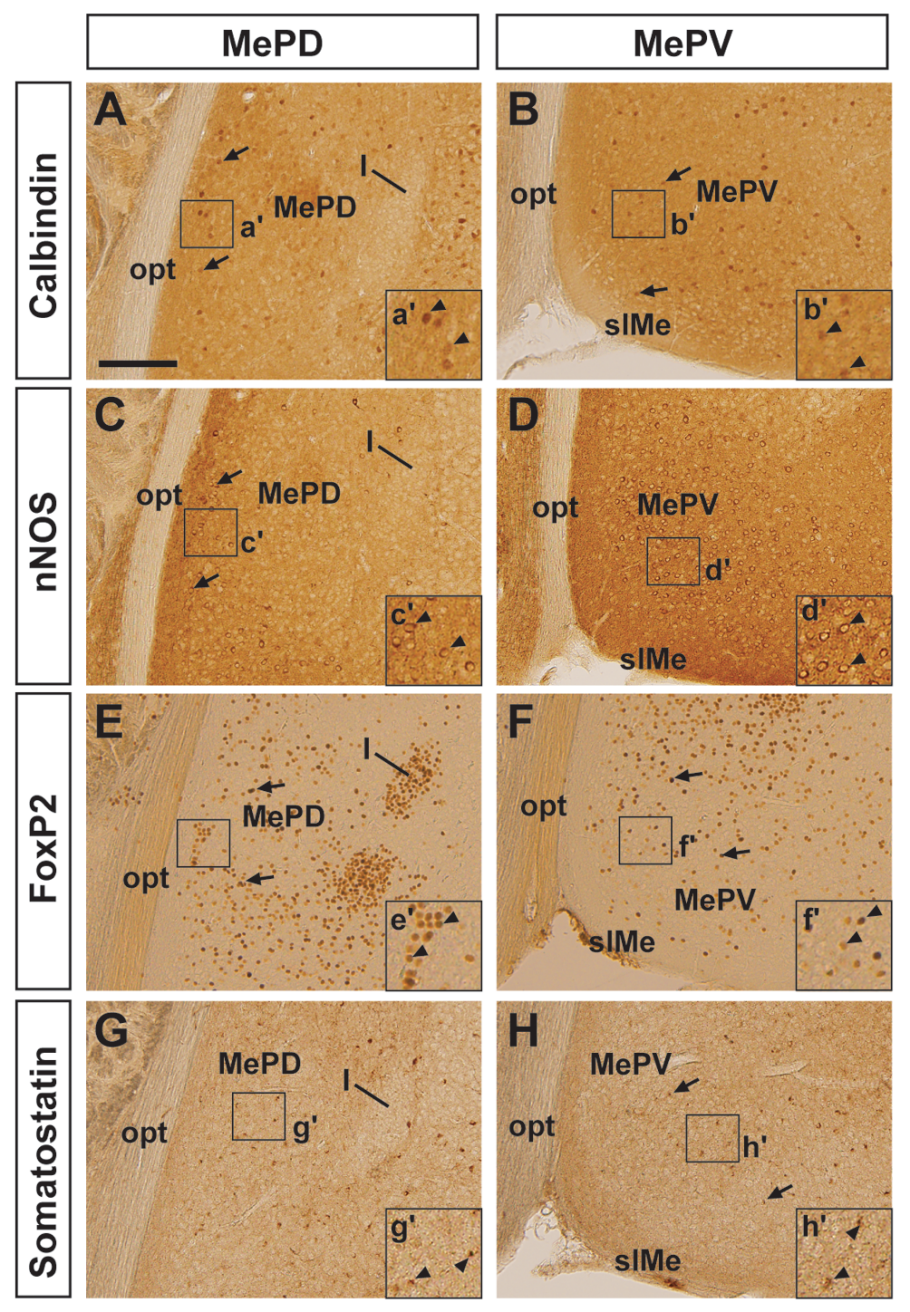
**Expression patterns of inhibitory neuronal markers in the MePD and MePV**. **(A-H) **Permanent immunohistochemistry in P22 wild-type brains (*n *= 3) showing the expression of classic inhibitory neuronal markers in the MePD (A,C,E,G) and MePV (B,D,F,H) of the posterior MeA. Calbindin (A,B, arrows), neuronal nitric oxide synthase (nNOS) (C,D, arrows), FoxP2 (E,F, arrows) and Somatostatin (G,H, arrows) expression was observed in both nuclear subdivisions of the posterior MeA. (a'-h') High-power views of boxed regions in corresponding panels. Abbreviations: I, intercalated nuclei of the amygdala, opt, optic tract; slME, superficial layer of the medial nucleus. Scale bar: 200 μm.

Interestingly, other markers showed differential expression patterns between both subdivisions. Calbindin, which labels approximately half of all cerebral cortical inhibitory neurons (reviewed in [32]), was observed scattered throughout the MePV, though as previously noted the distribution of Calbindin^+ ^cells appeared to be more concentrated near the optic tract (Figure 3A, B) [33]. Immunoreactivity to neuronal nitric oxide synthase (nNOS) labeled many medium-sized cells, which showed strong cytoplasmic staining surrounding an unstained nucleus and immunoreactive neuropil located in the MePV (Figure 3C), although similar to Calbindin, more nNOS^+ ^cells were observed in the medial part of the MePD, bordering the optic tract (Figure 3D). On the other hand, the expression of the Forkhead box transcription factor FoxP2, which marks a subpopulation of MeA neurons of unknown profiles [34], was highly expressed in both the main body of the MePV (Figure 3E), and the MePD (Figure 3F). We also observed that the interneuron marker Somatostatin (SST), which is expressed in the MeA [35], was found in scattered cells in both subdivisions (Figure 3G, H).

### *Nkx2-1-*, *Shh- *and *Gli1-*lineage cells contribute to inhibitory neuronal diversity in the posterior MeA

**Figure 4:**
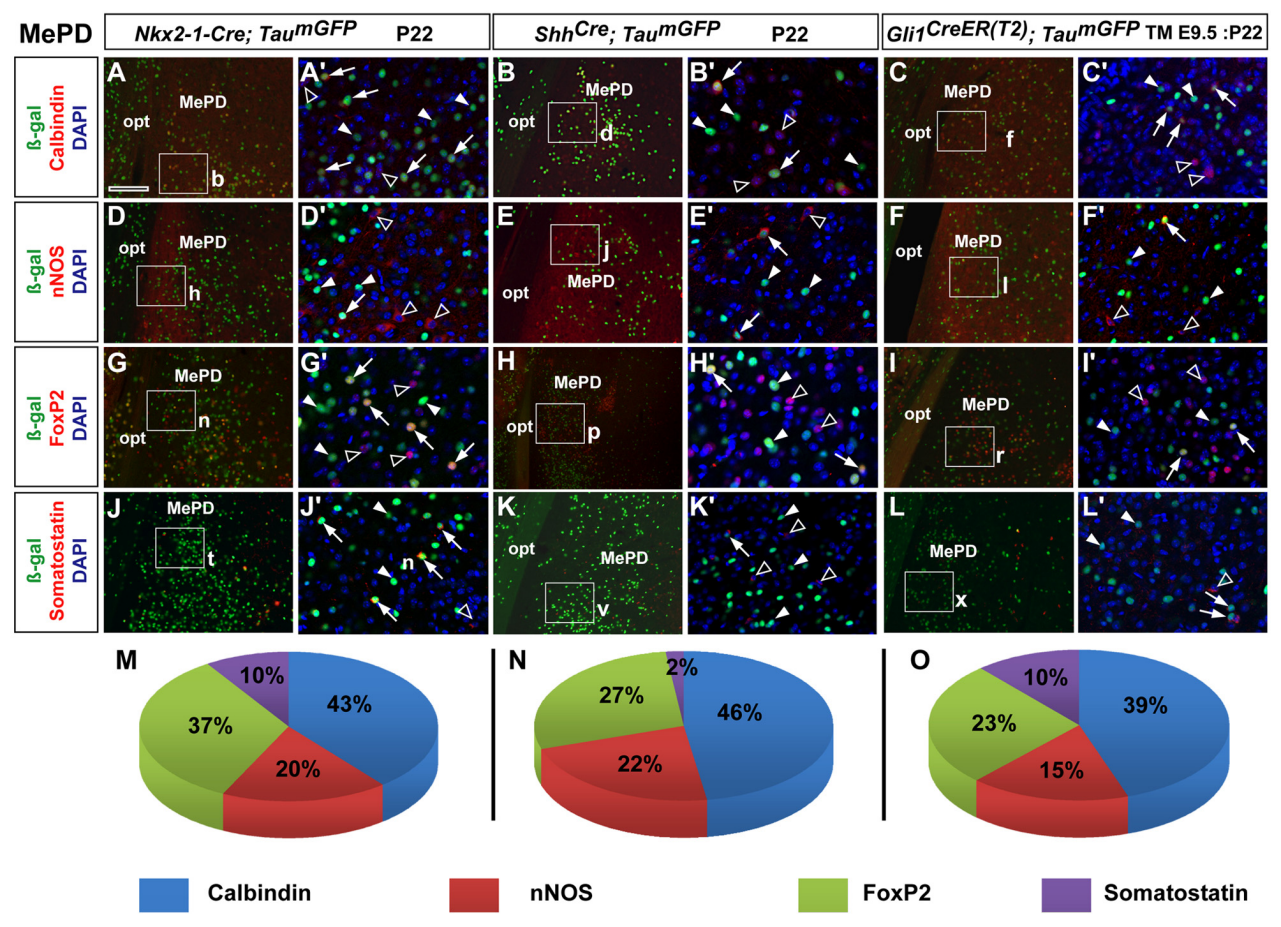
**Characterization of fate-mapped *Nkx2-1- *and *Shh*-lineage and *Shh*-responder cells in the postnatal medial posterodorsal nucleus**. **(A-L) **P22 coronal sections of the MePD from *Nkx2-1-Cre; Tau*^*mGFP *^(A,D,G,J), *Shh*^*Cre*^*; Tau*^*mGFP *^(B,E,H,K) and *Gli1*^*CreER(T2)*^*; Tau*^*mGFP *^(TM E9.5: P22) (C,F,I,L) brains showing dual immunofluorescence for β-galactosidase (β-gal^+^) recombined cells (green) and the expression of the inhibitory neuronal markers Calbindin (top row), neuronal nitric oxide synthase (nNOS; second row), FoxP2 (third row) and Somatostatin (last row). (A'-H') High-power images from corresponding boxed regions in (A-L) also show DAPI nuclear counterstaining (blue) in single optical sections from confocal images. Co-expressing cells (arrows), β-gal^+^/marker^- ^(closed arrowheads) and β-gal^-^/marker^+ ^(open arrowheads) cells are indicated. **(M-O) **Pie charts depicting the percentage value of β-gal^+ ^cells in each Cre line that co-expressed the corresponding neuronal marker. The images were taken from either the rostral or caudal portions of the posterior MeA in which the analysis was performed. Abbreviations: opt, optic tract. Scale bar: 200 μm (A-L); 100 μm (A'-L').

**Figure 5:**
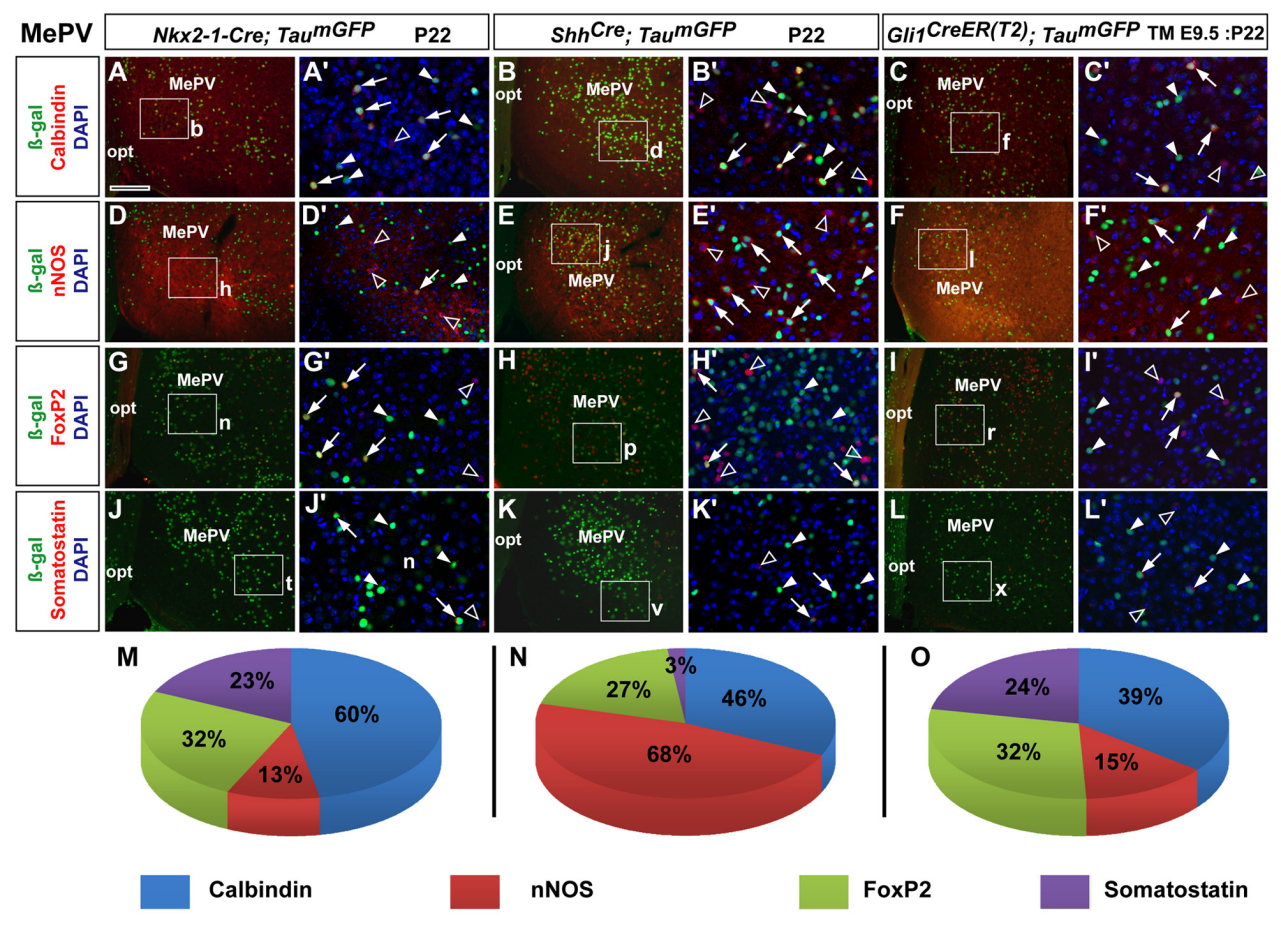
**Characterization of fate-mapped *Nkx2-1- *and *Shh*-lineage and *Shh*-responder cells in the postnatal medial posteroventral nucleus**. **(A-L) **P22 coronal sections of the MePV from *Nkx2-1-Cre; Tau*^*mGFP *^(A,D,G,J), *Shh*^*Cre*^*; Tau*^*mGFP *^(B,E,H,K) and *Gli1*^*CreER(T2)*^*; Tau*^*mGFP *^(TM E9.5: P22) (C,F,I,L) brains showing dual immunofluorescence for β-galactosidase (β-gal^+^) recombined cells (green) and the expression of the inhibitory neuronal markers Calbindin (top row), neuronal nitric oxide synthase (nNOS; second row), FoxP2 (third row) and Somatostatin (last row). **(A'-H') **High-power images from corresponding boxed regions in (A-L) also show DAPI nuclear counterstaining (blue) in single optical sections from confocal images. Co-expressing cells (arrows), β-gal^+^/marker^- ^(closed arrowheads) and β-gal^-^/marker^+ ^(open arrowheads) cells are indicated. **(M-O) **Pie charts depicting the percentage value of β-gal^+ ^cells in each Cre line that co-expressed the corresponding neuronal marker. The images were taken from either the rostral or caudal portions of the posterior MeA in which the analysis was performed. Abbreviations: opt, optic tract. Scale bar: 200 μm (A-L); 100 μm (A'-L').

Based on the above analysis of inhibitory neuronal distribution in the posterior MeA, we focused our fate analysis on the neuronal subtypes characterized by expression of Calbindin, nNOS, FoxP2, and SST. We performed immunolabeling for these markers in combination with an anti-β-galactosidase (β-gal) antibody to visualize nuclear staining in recombined cells from *Nkx2-1-Cre*, *Shh*^*Cre *^- and *Gli1*^*CreER(T2) *^(TM E9.5) brains (Figures 4 and 5). This analysis revealed that 69% of the β-gal^+ ^cells in the MePD were from the *Nkx2-1-Cre *lineage as opposed to 31% (3,733 versus 1,690 out of 5,423 cells, *n *= 2) from the *Shh*^*Cre *^lineage, which was highly statistically significant (*P *< 0.01). The MePV revealed the exact opposite as 69% of β-gal^+ ^cells were derived from the *Shh*^*Cre *^lineage and the *Nkx2-1-Cre *lineage contributed only 31% (3,132 versus 1,384 out of 4,516 cells, *n *= 2), which was also highly statistically significant (*P *< 0.01). From a single dose of tamoxifen administered at E9.5, in the inducible *Gli1*^*CreER(T2) *^line we observed that 61% of recombined cells (1,976 out of 3,265 β-gal^+ ^cells, *n *= 2), were located in the MePD. The contribution to the MePV, however, was lower (39%; 1,289 out of 3,265 β-gal^+ ^cells, *n *= 2), and significantly different (*P *< 0.01) from the MePD. Thus, consistent with the qualitative observations shown in Figure 2, these data reveal a significant differential distribution of *Nkx2-1*- and *Shh*-lineage cells in the MePD and MePV, respectively, with *Gli1*-derived cells showing a more significant contribution to the MePD.

Next, we analyzed the percentage co-expression of the markers Calbindin, nNOS, FoxP2 and SST in recombined cells in all three Cre lines. Calbindin was expressed by almost half of the recombined cells in the MePD of *Nkx2-1-Cre *(43 ± 13%, *n *= 740 β-gal^+ ^cells; Figure 4A, A'), *Shh*^*Cre *^(46 ± 16%, *n *= 350 β-gal^+ ^cells; Figure 4B, B') and *Gli1*^*CreER(T2) *^brains (39 ± 4%, *n *= 476 β-gal^+ ^cells; Figure 4C, C'). As mentioned previously, nNOS is expressed in a relatively low number of cells in the MePD compared to the MePV. Interestingly, for the MePD, the contributions of the *Nkx2-1 *lineage (20 ± 10%, *n *= 1,200 β-gal^+ ^cells; Figure 4D, D') and the *Shh-*lineage (22 ± 3%, *n *= 432 β-gal^+ ^cells; Figure 4E, E') to the nNOS population were remarkably similar. This likely reflects the contribution of the preoptic domains pPOA1 and pPOA2 in which *Nkx2-1 *and *Shh *are co-expressed [19] and is consistent with previous work from our laboratory showing that *Dbx1*^+ ^cells from the POA generate nNOS^+ ^cells in the MeA [3]. Furthermore, from early-labeled *Gli1*^*CreER(T2) *^(TM E9.5) brains we found that MePD nNOS^+ ^cells also derive from this *Shh*-responding population (15 ± 6%, *n *= 621 β-gal^+ ^cells; Figure 4F, F'). In addition, FoxP2^+ ^cells were derived from both *Nkx2-1 *(37 ± 10%, *n *= 903 β-gal^+ ^cells; Figure 4G, G') and *Shh *(27 ± 12%, *n *= 477 β-gal^+ ^cells; Figure 4H, H') lineages in the MePD, although this difference was not statistically significant (*P *= 0.48). *Gli1*-derived cells co-expressing FoxP2 were abundant in the MePD (23 ± 6%, *n *= 450 β-gal^+ ^cells; Figure 4I, I'), showing that progenitor cells that respond to *Shh *signaling from an early developmental stage generate this inhibitory cell type in the posterior MeA. Interestingly, in the MePD we found a statistically significant (*P *< 0.01) difference in the generation of SST-positive cells from the *Nkx2-1*-lineage cells (10 ± 1%, *n *= 1116 β-gal^+ ^cells; Figure 4J, J') versus the *Shh*-lineage cells (2 ± 2%, *n *= 431 β-gal^+ ^cells; Figure 4K, K'). SST^+ ^cells were also significantly generated from *Gli1*-expressing progenitors (20 ± 10%, *n *= 590 β-gal^+ ^cells; Figure 4L, L'), which has also been observed for the cerebral cortex [36].

**Figure 6:**
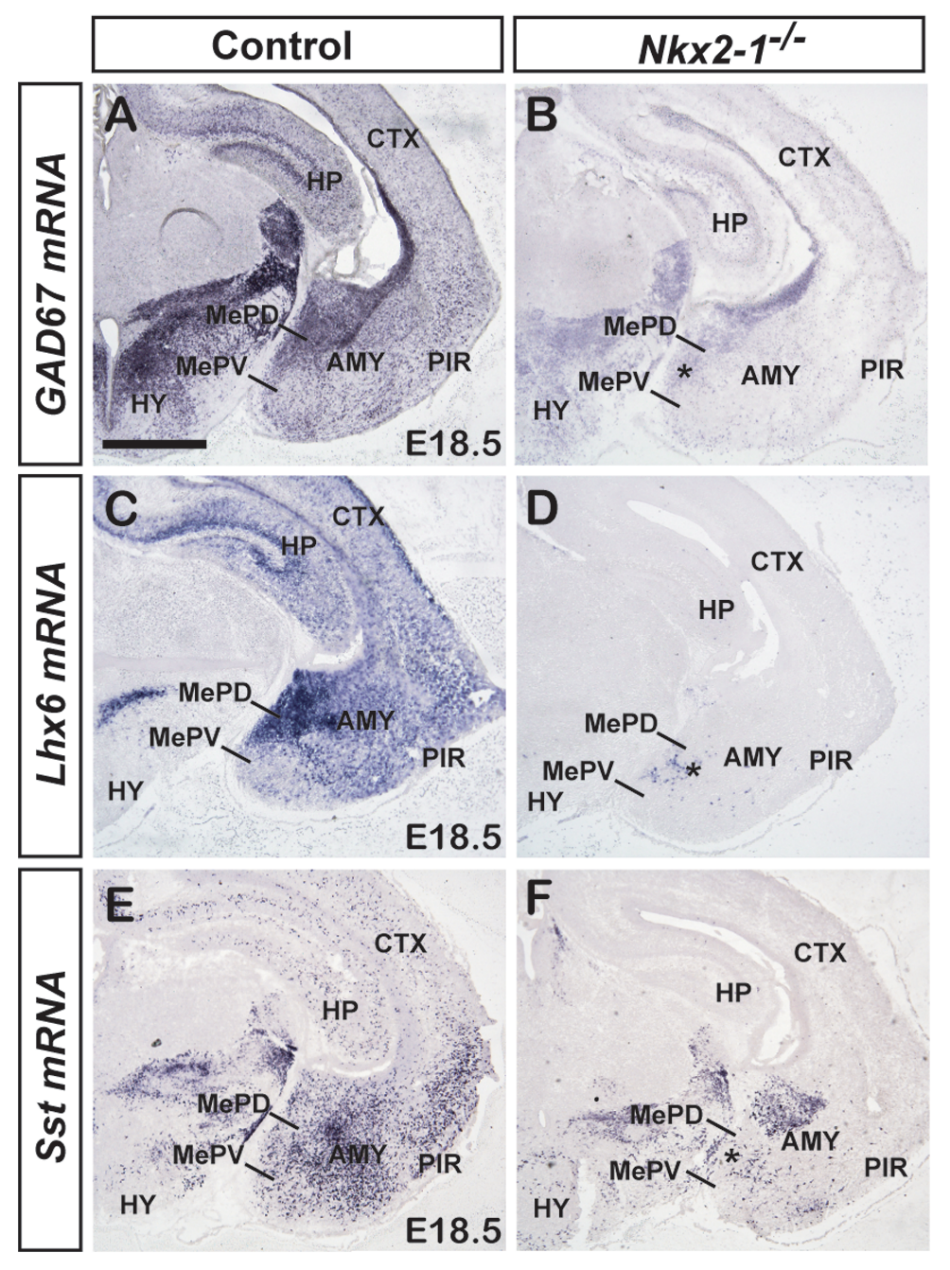
**Major defects in mRNA expression of putative inhibitory neuronal subtypes in the MePD of *Nkx2-1 *mutant mice**. **(A-F) **mRNA expression in E18.5 control (A,C,E) or *Nkx2-1 *mutant (B,D,F) posterior MeA reveals significant defects in inhibitory neuronal subtypes, particularly in the MePD. (A,B) The mRNA expression of *GAD67*, a pan-inhibitory marker, is severely reduced in *Nkx2-1 *mutant mice (B) compared to controls (A). This is particularly evident in the MePD of the mutant (B, asterisk) as *GAD67 *mRNA expression appears stronger in the MePD in controls (A). (C,D) The *Lhx6 *mRNA expression in controls (C) is similar to that observed for *GAD67*, being notably stronger in the MePD than MePV. Although it is known that *Nkx2-1 *directly regulates *Lhx6 *expression, remnant *Lhx6 *mRNA expression was observed in the mutant posterior MeA, particularly around the MePD (D, asterisk). (E,F) The same was observed for *Sst *mRNA, whereby the expression in the control MePD (E) was significantly reduced in the mutant (F), although some remnant expression of *Sst *was observed in the MePD (F, asterisk). *n *= 4 for both control and *Nkx2-1 *mutant for each gene. Abbreviations: AMY, amygdala; CTX, cortex; HP, hippocampus; HY, hypothalamus; PIR, piriform cortex. Scale bar: A-F: 500 μm.

In the MePV, we also found that recombined cells from all three Cre lines showed high co-expression of Calbindin, indicating an inhibitory neuronal phenotype. As expected, a large number of recombined cells from the *Nkx2-1 *lineage co-expressed Calbindin (60 ± 14%, *n *= 282 β-gal^+ ^cells; Figure 5A, A'), although the percentage values determined from *Shh*^*Cre *^(46 ± 11%, *n *= 761 β-gal^+ ^cells; Figure 5B, B') and *Gli1*^*CreER(T2) *^(TM E9.5) brains (48 ± 9%, *n *= 300 β-gal^+ ^cells; Figure 5C, C') were slightly lower, but similar to the MePD values. As shown earlier, nNOS expression between the two nuclei is disproportionately higher in the MePV (Figure 6), which is preferentially attributed to the *Shh*-lineage cells. Accordingly, we found that *Shh*^*Cre *^recombined cells had high co-expression with nNOS (68 ± 11%, *n *= 884 β-gal^+ ^cells; Figure 5E, E'), whereas *Nkx2-1*-lineage cells generated a much lower proportion of nNOS^+ ^cells (13 ± 6%, *n *= 331 β-gal^+ ^cells; Figure 5D, D'), which was statistically significant (*P *< 0.05) between both groups. The *Shh*-responding cell population also co-expressed nNOS (29 ± 11%, *n *= 461 β-gal^+ ^cells; Figure 5F, F') in the MePV. Similar to the MePD, all recombined cells from both genetic lineages co-expressed FoxP2 in the MePV. Likewise, a higher percentage of co-localization was found in the *Nkx2-1-Cre *(32 ± 10%, *n *= 357 β-gal^+ ^cells; Figure 5G, G') than the *Shh*^*Cre *^brains (27 ± 13%, *n *= 940 β-gal^+ ^cells; Figure 5H, H'), although this did not reach statistical significance. A similar percentage co-expression was observed in the *Gli1*^*CreER(T2) *^(TM E9.5) brains (24 ± 10%, *n *= 310 β-gal^+ ^cells; Figure 5I, I'). Finally, we quantified the occurrence of β-gal/SST double positive cells in the MePV. Interestingly, we also found a preferential bias for the *Nkx2-1 *lineage to generate SST^+ ^cells in the MePV (23 ± 4%, *n *= 414 β-gal^+ ^cells; Figure 5J, J'), as apposed to the *Shh*^*Cre *^lineage (3 ± 2%, *n *= 547 β-gal^+ ^cells; Figure 5K, K'). *Gli1*-derived cells also had high co-expression with SST (24 ± 10%, *n *= 310 β-gal^+ ^cells; Figure 5L, L').

In summary, these data revealed that: *Nkx2-1*- and *Shh*-lineage cells show a complementary distribution between the dorsal and ventral subdivisions in the adult posterior MeA - in contrast, *Gli1*-derived neurons contribute more predominantly to the MePD but heavily to both subdivisions; nNOS^+ ^cells, which are preferentially localized in the MePV, are primarily derived from *Shh*-lineage cells; *Nkx2-1*-lineage cells generated a higher proportion of SST^+ ^cells in the MePD and MePV than *Shh*-lineage cells; *Nkx2-1*-derived cells showed a higher co-expression with FoxP2 than *Shh*-lineage cells, although within both lineages there was no discrimination between the MePV and the MePD; and early-generated *Gli1*^+ ^progenitor cells generated inhibitory neuronal cells in proportions similar to those derived from the *Nkx2-1 *lineage.

### *Nkx2-1 *mutant analysis demonstrates a greater functional role for *Nkx2-1 *in the MePD compared to the MePV

Having shown that the *Nkx2-1 *lineage contributes to neural diversity in the posterior MeA, we next sought to determine whether there is a functional requirement for *Nkx2-1 *in the generation of these cell types. To this end, we analyzed the expression of genes that mark the above-described inhibitory neurons in *Nkx2-1 *mutant mice [37]. This analysis was accomplished at E18.5, the latest stage possible due to perinatal lethality. First, we used *GAD67 *mRNA expression as a pan-inhibitory neuronal marker, which in controls is abundantly expressed throughout the whole telencephalon, including the hippocampus, cortex, striatum and amygdala (Figure 6A). In agreement with prior studies [18,38,39], we observed a major reduction in *GAD67 *mRNA expression throughout the *Nkx2-1 *mutant telencephalon, including the posterior MeA (Figure 6B). In particular, the MePD displayed intense *GAD67 *mRNA expression in the control (Figure 6A), which was reduced in the absence of *Nkx2-1 *(Figure 6B). The mutant MePV also showed a clear reduction in *GAD67 *mRNA expression compared to control (Figure 6A, B). Next, we examined the expression of *Lhx6*, which, similar to *GAD67*, exhibited notably more intense expression in the MePD than the MePV in control brains (Figure 6C). Prior studies have described a complete loss of *Lhx6 *in the *Nkx2-1 *mutant telencephalon [18,40], which was later explained by the demonstration that *Nkx2-1 *directly regulates *Lhx6 *transcription [17]. Therefore, we were surprised to find a subset of cells in the posterior MeA that expressed *Lhx6 *mRNA in the *Nkx2-1 *mutant (Figure 6D). These 'remnant' *Lhx6 *mRNA-expressing cells were located in the MePD, whereas the MePV was devoid of *Lhx6 *expression (Figure 6D). Subsequently, we examined *Sst *mRNA expression. In control, there were numerous *Sst *mRNA-expressing cells in the MePD, whereas the MePV contained only a few positive cells (Figure 6E). However, similar to *Lhx6*, we observed a number of remaining *Sst *mRNA-expressing cells that were primarily observed in the MePD (Figure 6F). This is in interesting contrast to other areas of the telencephalon, such as the cerebral cortex and hippocampus, that display a complete absence of *Sst *mRNA-expressing cells. A large number of *Sst *mRNA-expressing cells in the central nucleus was also maintained in the absence of *Nkx2-1 *(Figure 6F).

This analysis demonstrates that *Nkx2-1 *plays an important role in the specification of inhibitory neuronal subtypes in the posterior MeA. Surprisingly, however, we noticed that a population of cells that expressed *Lhx6 *and *Sst *mRNA persisted in the MePD in the *Nkx2-1 *mutant. Therefore, in interesting contrast to the cerebral cortex, a small population of posterior MeA *Lhx6*^+ ^and *Sst*^+ ^cells is not dependent on *Nkx2-1 *function.

**Figure 7:**
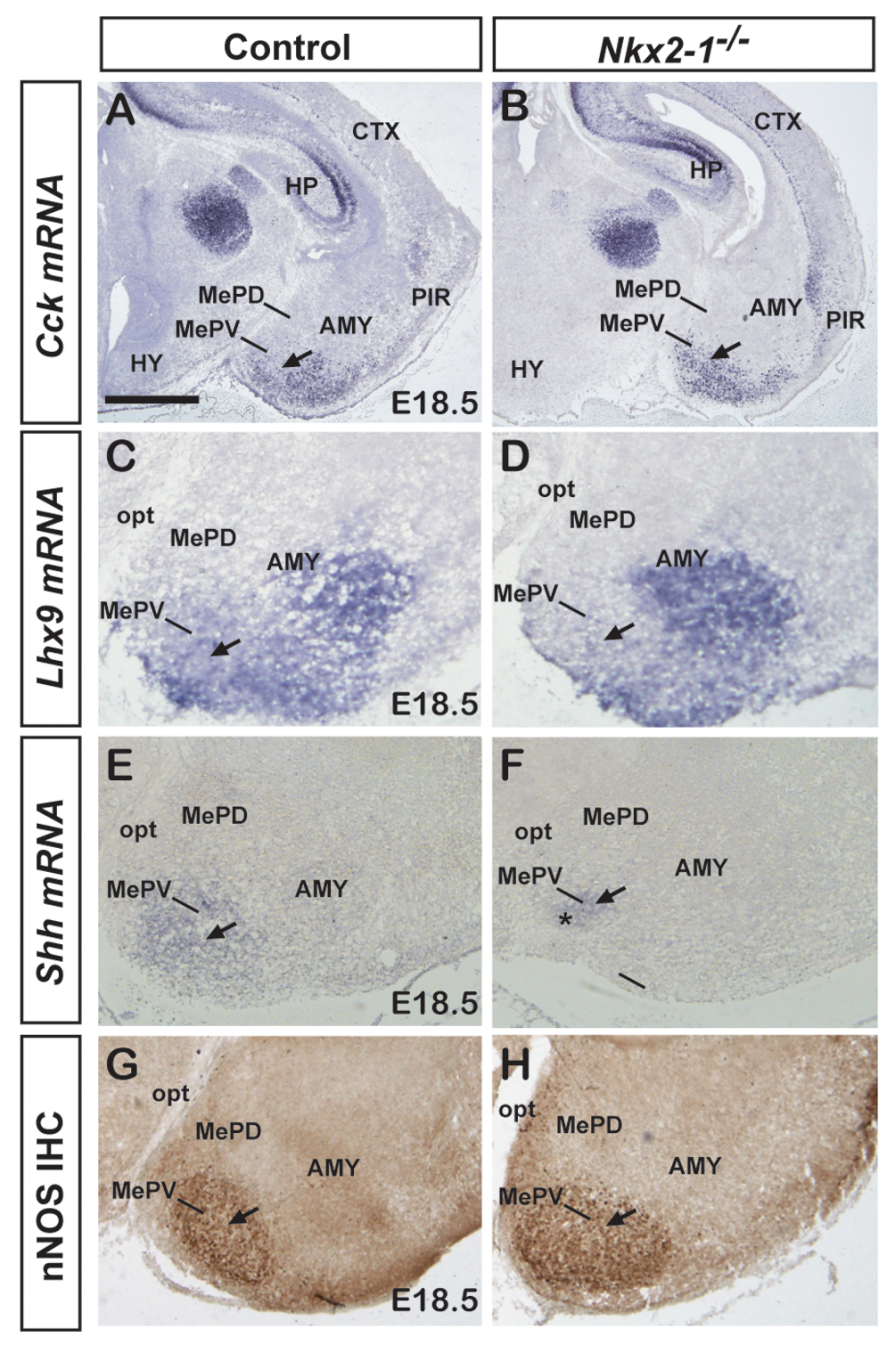
**Subtle defects in mRNA and protein expression of putative inhibitory neuronal subtypes in the MePV *of Nkx2-1 *mutant mice**. **(A-H) **mRNA and protein expression in E18.5 control (A,C,E,G) or *Nkx2-1 *mutant (B,D,F,H) posterior MeA reveals subtle or no apparent defects in inhibitory neuronal subtypes in the MePV. (A,B) The mRNA expression of *Cck *does not appear to be altered in *Nkx2-1 *mutant mice (B, arrow) compared to control (A, arrow). (C,D) *Lhx9 *mRNA expression, which in controls is found in the MePV (C, arrow) is observed in the mutant MePV (D, arrow), although the expression appears to be weaker. (E,F) *Shh *mRNA expression, which is found in the control MePV (E, arrow), is maintained in the absence of *Nkx2-1*, albeit in a reduced expression domain (F, arrow). (G,H) Permanent immunohistochemistry for nNOS shows a large cluster of immunopositive cells in both the control (G, arrow) and mutant MePV (H, arrow), indicating that *Nkx2-1 *does not play a significant role in specification or maintenance of MePV cell populations. *n *= 3 for both control and *Nkx2-1 *mutant for each gene and for nNOS immunohistochemistry. Abbreviations: AMY, amygdala; CTX, cortex; HP, hippocampus; HY, hypothalamus; opt, optic tract; PIR, piriform cortex. Scale bar: 500 μm (A,B); 250 μm (C-F); 300 μm (G,H).

To further explore the potential differential function of *Nkx2-1 *in the MePD and MePV, we examined the expression of genes that specifically mark the MePV, such as *Cck*, *Shh*, *Lhx9 *and nNOS in *Nkx2-1 *mutants (Figure 7). In the absence of *Nkx2-1*, there was no noticeable change in *Cck *mRNA expression in the mutant MePV compared to control (Figure 7A, B). Next, we analyzed *Lhx9*, which is expressed in the embryonic and postnatal MePV [2,12,41]. Previous studies have shown that *Lhx9 *mRNA expression is restricted to the superficial layer of the posterior MeA and the MePV [41]. In our hands, a prolonged exposure (up to 48 hours) revealed a signal, albeit weak, in the body of the MePV in both control and mutant brains. When compared to *Lhx9 *mRNA expression in the control MePV (Figure 7C), expression in the mutant was present but appeared weak (Figure 7D), possibly indicating a subtle effect by the absence of *Nkx2-1*. We next examined the expression of *Shh*, which is expressed in the embryonic MePV [2]. Previously it was shown that at earlier developmental stages in the *Nkx2-1 *mutant, most telencephalic expression of *Shh *mRNA is lost, with the exception of a small domain caudally that corresponds to the prospective amygdala [23]. When examined at E18.5, the expression of *Shh *mRNA that is observed in the control MePV (Figure 7E) is maintained in the mutant MePV (Figure 7F), although the expression domain is significantly smaller. Lastly, we examined the expression of nNOS, where expression is notably higher in the MePV than the MePD [33]. Permanent immunohistochemistry for nNOS showed strong expression in the control MePV (Figure 7G), which, interestingly, does not appear to be significantly altered in the *Nkx2-1 *mutant (Figure 7H).

In summary, the *Nkx2-1 *mutant analysis at E18.5 suggests a functional role for this gene in both the MePD and MePV, with primary effects on the MePD. This is consistent with the fate-mapping analysis (Figure 2) in which *Nkx2-1*-lineage cells show a major contribution to the MePD.

### Electrophysiological characterization reveals three distinct functional classes of *Shh-*lineage neurons in the posterior MeA

**Figure 8:**
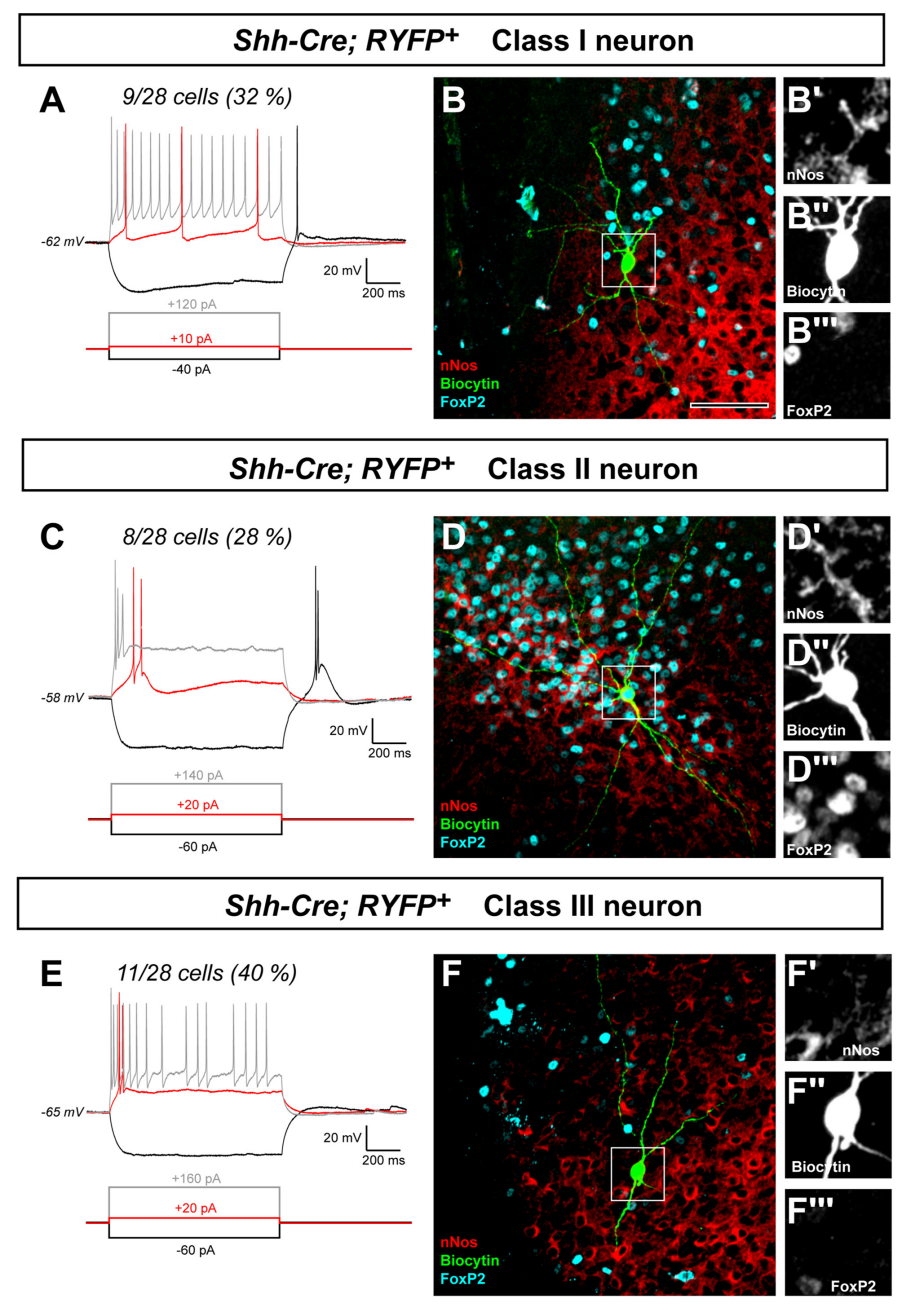
**Electrophysiological characterization reveals three functionally distinct classes of *Shh*-lineage cells in the posterior medial amygdala**. **(A-F) **Representative examples of three classes of *Shh*^*Cre*^*; RYFP*^*+ *^neurons recorded by patch-clamp in current-clamp mode (A,C,E), filled with biocytin (B,D,F; green) and immunostained for nNos (B,D,F; red) and FoxP2 (B,D,F; blue). **(B'-B''', D'-D''',F'-F''') **Single optical sections from high-power confocal imaging of biocytin, nNOS or FoxP2 immunohistochemistry. Each neuron was recorded at its resting membrane potential, as given at the left of each group of traces. Neurons were subjected to a hyperpolarizing step (A,C,E; black) to test for the presence of sag current and rebound firing, a small depolarizing step corresponding to the rheobase of the neuron (A,C,E; red) and the minimal depolarizing step able to induce firing at a maximum frequency (A,C,E; grey). Current injection step values are indicated in the bottom of each recording and accordingly colored. The fraction and percentage of *Shh*^*Cre*^*; RYFP*^*+ *^neurons corresponding to each class are indicated on the top of each group of traces. Scale bar: 80 μm (A-H); 40 μm (B'-E''').

The overlapping pattern of *Shh *and *Dbx1 *expression in the embryonic POA [19], and the remarkable similarity in distribution and high percentage of nNOS co-localization between *Dbx1*-derived [3] and *Shh*-derived neurons (Figure 5) raised the question as to whether there were functional differences in these two lineages. To examine this we performed an electrophysiological characterization of *Shh*-lineage populations in the posterior MeA. In order to visualize the recombined cells, we crossed *Shh*^*Cre *^mice with the *RYFP *reporter line, which our group has previously found to facilitate electrophysiological analysis [3,8]. Importantly, the distribution of recombined *Shh*^*Cre *^cells in the *RYFP *reporter line was identical to that of the *Tau*^*mGFP *^reporter line (Additional file 2A, B). We carried out whole-cell patch clamp recordings of recombinant YFP^+ ^neurons from *Shh*^*Cre*^*; RYFP *mice at P17 to P23. Each recorded neuron was filled with biocytin and slices were subsequently fixed and immunostained for nNOS and FoxP2. From this analysis, we could discriminate three populations of YFP^+ ^neurons that exhibited distinct responses to a hyperpolarizing current injection, and also differed in their firing pattern and immunohistochemical profile (Figure 8; Additional file 3).

When hyperpolarized to -100 mV by injecting a step current (-40 to 120 pA, 1,000 ms), the first class of YFP^+ ^neurons (class I; 9 out of 28 neurons) was characterized by clear depolarizing sag current (5.5 ± 2 mV; *n *= 9), usually resulting in a single rebound spike upon repolarization (*n *= 7 out of 9; Figure 8A). A small depolarizing step current at rheobase value (10 to 40 pA, 1,000 ms) typically induced a series of three to four spikes separated by relatively large and slow rising afterhyperpolarization (amplitude = -11.2 ± 2.5 mV; decay half-time = 50.7 ± 14.7 ms). Larger depolarizing current steps (100 to 200 pA, 1,000 ms) were injected to determine their maximal discharge frequency (24 ± 5 Hz) and showed accommodating firing patterns (accommodation ratio = 0.46 ± 0.08). *Post hoc *immunohistochemical analysis showed that all neurons in this class were nNOS^+^/FoxP2^- ^(Figure 8B), with the exception of one cell that was immunonegative for both markers. Interestingly, both the electrophysiological and immunohistochemical profile of this first class of YFP^+ ^neurons made them indistinguishable from nNOS^+ ^MeA neurons generated from the *Dbx1*-derived progenitors previously described by our laboratory [3].

A second class of YFP^+ ^neurons (class II; 8 out of 28 neurons) could be distinguished by their inability to repetitively fire even when large depolarizing currents were injected (100 to 300 pA, 1,000 ms) (Figure 8C). These neurons exhibited a single or dual spike discharge upon depolarization. At rheobase value (20 to 80 pA), the discharge systematically rode on a slow-depolarizing envelope (amplitude = 18 ± 8 mV), suggesting the presence of a low-threshold Ca^++ ^current (I_T_). These neurons were exempt of sag currents when hyperpolarized to -100 mV but would exhibit a slow-depolarizing envelope upon repolarization, confirming the likely presence of large I_T _currents in these cells. Interestingly, all of these neurons were FoxP2^+ ^and most were also nNOS^+ ^(6 out of 8 cells; Figure 8D).

The third class of YFP^+ ^neurons (class III; 11 out of 28 neurons) was characterized by an irregular firing (frequency 5 to 20 Hz) in response to a large depolarization (Figure 8E). At rheobase value (20 to 80 pA), these neurons displayed a single early discharge of one to three spikes riding a small depolarizing envelope (10 ± 5 mV) and exhibited sharp fast-rising afterhyperpolarization (amplitude = -13.2 ± 1.5 mV; decay half-time = 15.4 ± 4.2 ms). In response to larger depolarizing current steps (100 to 200 pA, 1,000 ms), these cells exhibited a very variable maximal discharge frequency (11 ± 5 Hz) ranging from a single initial burst of three to five spikes to a series of two or three bursts of three to five spikes each. When hyperpolarized to -100 mV, these cells exhibited no or a minimal sag current (1.5 ± 0.8 mV) and small rebound I_T_-like currents were observed (10 ± 5 mV). In most cells (9 out of 11 cells) these currents were not large enough to induce a rebound spike. All cells in this third class of cells were immunonegative for both nNOS and FoxP2 (Figure 8F).

Taken together, the results from the electrophysiological and *post hoc *immunohistochemical analyses revealed that *Shh-*lineage neurons generate functionally distinct inhibitory neuronal classes in the posterior MeA. We find that in addition to the class I neurons that are similar to neurons derived from the *Dbx1*^*+ *^lineage [3], at least two other classes of neurons have distinct electrophysiological and molecular profiles. Based on their expression of nNOS, a marker of MeA inhibitory projection neurons, as well as their morphology, we speculate that the classes I and II are inhibitory projection neurons. Our electrophysiological analysis shows that our *Shh*-lineage class III neurons that lack expression of both nNOS and FoxP2 exhibit electrophysiological characteristics that partially reflect a glutamatergic phenotype in the posterior MeA, which has been suggested by a prior study [42]. However, the restriction of these neurons to a Tbr1-negative region indicates that they may possess inhibitory character. Interestingly, we found that *Shh*-lineage cells gave rise to a subset of deep layer neurons in the cerebral cortex with a pyramidal morphology (data not shown), indicating that these progenitors can give rise to a subset of glutamatergic neurons. However, the vast majority of *Shh*-lineage cells in the amygdala are likely to be inhibitory because the MeA is primarily GABAergic in neuronal output, as shown by intense expression of GAD65 and GAD67 ([1] and this study) and because nNOS [43] and Calbindin, which are expressed in most *Shh*-lineage neurons, are markers of inhibitory neurons.

## Discussion

**Figure 9:**
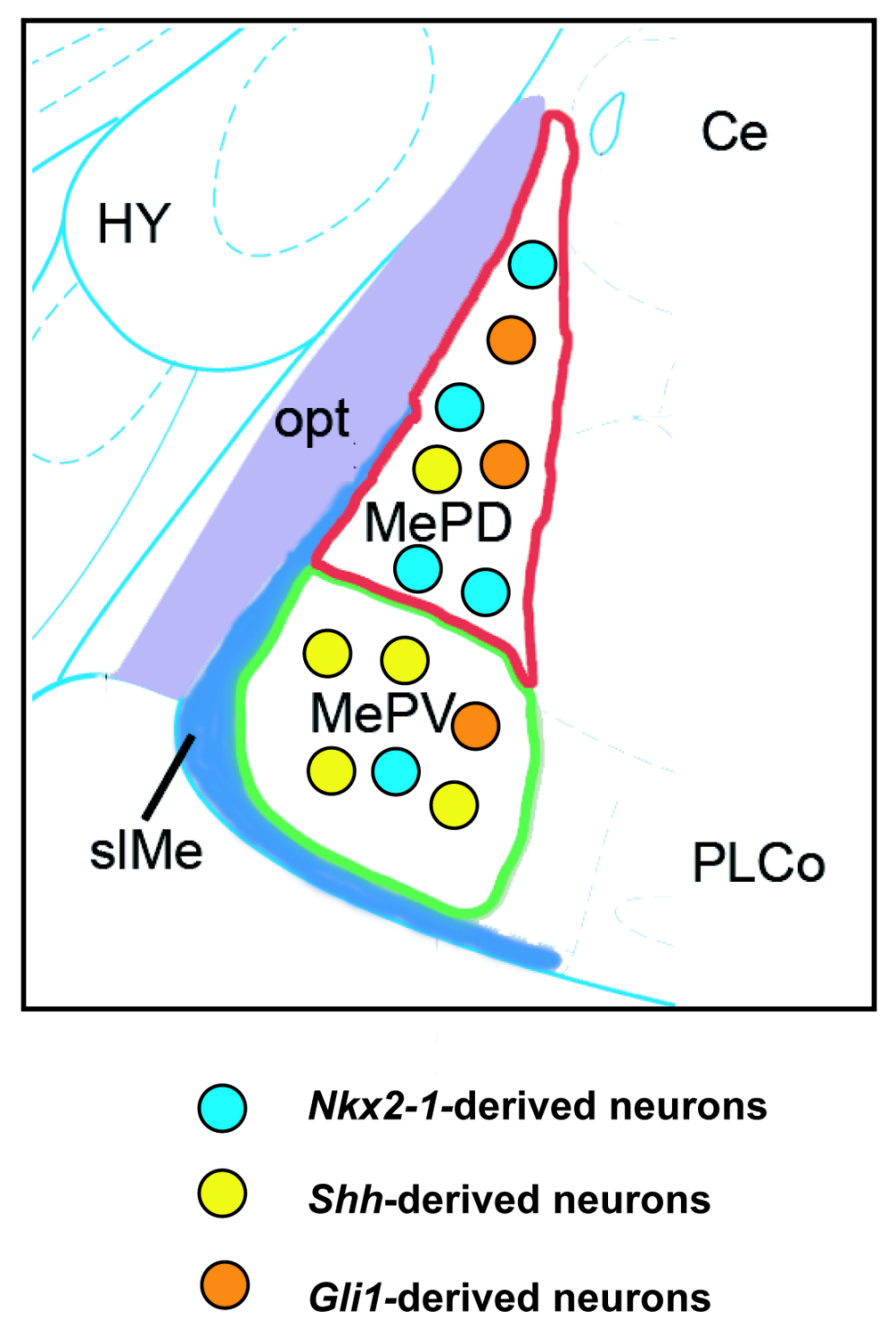
**Schema *of Nkx2-1-*, *Shh*- and *Gli1*-derived neurons in the dorsal and ventral subdivisions of the posterior medial amygdala**. Schema summarizes the fate-mapping data. *Nkx2-1*-derived neurons (blue colored circles) preferentially fate-mapped to the MePD (MePD, 69%; MePV, 31%). *Shh*-derived neurons (yellow colored circles) were distributed in an exact mirror-image pattern (MePD, 31%; MePV, 69%). Similar to *Nkx2-1*-derived neurons, *Gli1*-derived neurons (orange colored circles) were found predominantly in the MePD (61%) compared to the MePV (39%). The differential inhibitory neuronal subtype fates are summarized in the pie charts in Figures 4M,N,O and 5M,N,O. Abbreviations: Ce, central nucleus; HY, hypothalamus; opt, optic tract; PLCo, posterolateral cortical amygdala nucleus; PRh, perirhinal cortex; slMe, superficial layer of the medial nucleus.

Genetic fate-mapping is a powerful tool to elucidate the contribution of progenitor populations marked by individual genes to the cellular diversity found within the adult mammalian forebrain. Using this approach, we correlated the neurochemical phenotypes in the postnatal amygdala with defined domains of gene expression in the embryonic forebrain. With regard to the amygdala, genetic fate-mapping thus far has shown that excitatory neurons in the basolateral complex are derived from the pallial *Emx1 *lineage and the ventral pallial *Dbx1 *lineage [3,8,44]. In addition, pan-subpallial fate-mapping using the *Dlx5/6 *enhancer shows substantial recombination in the medial and central nuclei as well as the basolateral complex [45], consistent with subpallial sources of amygdala local interneurons and inhibitory projection neurons. Genes with more restricted expression patterns have shown that the subpallial embryonic POA, which expresses *Dbx1 *and *Nkx5-1*, contributes to neural diversity in the MeA [3,46]. Here, in agreement with a prior study [5], we show that cells expressing the transcription factor *Nkx2-1*, which is expressed in the embryonic MGE and POA [18,19], also contributes to inhibitory neuronal diversity in the MeA. Importantly, we significantly extend this knowledge by showing: 1, that *Nkx2-1-*lineage cells have a biased contribution to the dorsal subdivision of the posterior MeA (MePD); 2, consistent with this, *Nkx2-1 *functions to a greater degree in the development of the MePD as opposed to the MePV; 3, *Shh-*lineage cells generate complementary patterns of cellular diversity in the ventral subdivision of the posterior MeA, with a greater bias toward generation of MePV neurons; 4, *Shh*-responding (*Gli1*^*CreER(T2)*^^+^) progenitor cells contribute to posterior MeA neural diversity from approximately E10.5 (Figure 9); and 5, neurons derived from the *Shh*-lineage are a functionally diverse group consisting of at least three classes of neurons as defined from unique combinations of electrophysiological and neurochemical profiles.

### *Shh- *and *Nkx2-1-*lineage cells generate distinct classes of inhibitory neurons in the posterior MeA

*Shh *is the most studied of the *Hedgehog *family of secreted glycoproteins and is a potent morphogen that plays a crucial role in ventral patterning of many tissues, including the forebrain, midbrain, cerebellum, spinal cord and the limb (reviewed by [47-52]). Here, we used a Cre line under the transcriptional regulation of *Shh *[22] to indelibly label *Shh-*lineage (*Shh*^*Cre*^^+^) cells from early embryonic development through to adulthood. Using this genetic fate-mapping tool we were able to identify a predominant contribution of *Shh*-expressing cells in the generation of inhibitory neural diversity in the MeA. Specifically, we show that *Shh*-lineage cells preferentially generate the cells in the MePV, which express nNOS (68% of recombined cells), a marker of hypothalamic projecting inhibitory output neurons [43]. Interestingly, a subset of these *Shh*-lineage, nNOS^+ ^cells (the class I type) display electrophysiological and molecular signatures reminiscent of our previously identified *Dbx1*-derived MeA neurons [3]. Therefore, this subpopulation likely derives from the ventral subdivision of the POA (pPOA2), which expresses *Dbx1 *[3]. Thus, similar to the generation of midbrain dopaminergic neurons [53], *Shh*-lineage cells appear to give rise to diverse neural subtypes in the telencephalon.

The co-expression of FoxP2 with nNOS in the class II *Shh*-derived neurons is also quite intriguing. FoxP2 has been identified as essential for development of brain circuitry involved in speech in humans and vocalization in other vertebrates. Mutations in FoxP2 have also been implicated in both language disorders and autism, the latter of which is characterized by both language and amygdala-based impairments (reviewed in [54,55]). Therefore, it is possible that the electrophysiological and marker diversity of *Shh*-lineage neurons underlies important differences in both pathways of connectivity and function of MeA output neurons. For example, as the MeA is a central component of circuitry that regulates innate behaviors such as feeding, reproduction, aggression and maternal bonding, perhaps these different *Shh*-lineage populations differentially modulate these behaviors. Developmentally, it is also possible that transcription factor genes that show discrete and overlapping expression domains within the POA, such *Dlx1*, *Nkx6-2*, *Nkx5-1 *or *Lhx2 *[19,46], may selectively or combinatorally generate the class I to III electrophysiological subtypes that we describe here. In addition, as *Shh *and *Dbx1 *are expressed in the embryonic hypothalamus [23,56,57], which itself has been speculated to generate cells destined for the amygdala [4,6], ], further complexity or refinement of neural populations may be related to more precise origins within the developing forebrain.

We also find that, in contrast to *Shh*-lineage neurons, *Nkx2-1*-derived cells preferentially fate-map to the MePD, a nucleus that regulates reproductive behaviors. A previous study showed that *Nkx2-1*-lineage cells generate inhibitory cells in the MeA [5], in addition to the known and recently identified roles for *Nkx2-1 *in generating inhibitory cells for the cerebral cortex [5,18,58] and globus pallidus [29,59], respectively. We find that analogous to the cerebral cortex, a number of SST^+ ^cells are *Nkx2-1*-derived, although another subset are derived from *Shh*-lineage progenitor cells. *Nkx2-1*-lineage cells show high co-expression with Calbindin and FoxP2 in both the MePD and MePV. Whereas *Shh*-lineage cells generate the vast majority of nNOS^+ ^cells in the MePV, *Nkx2-1*-lineage cells generate an equivalent portion of nNOS^+ ^cells within both nuclei. Therefore, this study reveals that inhibitory neural diversity in the posterior MeA is generated from *Shh-*expressing and *Shh*-responding (*Nkx2-1-Cre*^*+ *^and *Gli1*^*CreER(T2)+*^) components of the *Shh *signaling pathway (see Figure 9 for schematic).

### Function of *Nkx2-1 *in medial amygdala patterning

The transcription factor *Nkx2-1 *plays a major role in the generation of inhibitory neurons in the forebrain [5,18,60] and the expression of *Nkx2-1 *is largely dependent on *Shh *[61]. The notable exception to this is at the level of the prospective amygdala, where a small focal expression domain of *Shh *remains in *Nkx2-1 *mutants [23]. Interestingly, as previously shown, this remnant expression is coincident with the persistence of markers of the oligodendrocyte lineage [23]. Here, we show that *Shh*-lineage cells generate inhibitory neurons as shown by the differential expression of Calbindin, nNOS, FoxP2 and SST. Our gene expression analysis in *Nkx2-1 *mutant embryos revealed that *Nkx2-1 *has a significant functional role in the development of the MePD, where we observed major decreases in *GAD67*, *Lhx6 *and *Sst *mRNA gene expression. This finding is consistent with previous studies that have described a complete loss of *Lhx6 *mRNA expression in the *Nkx2-1 *mutant telencephalon [18,40]. Surprisingly, we observed a sparing of a subset of these *GAD67*^*+*^, *Lhx6*^*+ *^and *Sst*^*+ *^neurons in the MeA. As *Nkx2-1 *has more recently been shown to directly regulate *Lhx6 *transcription [17], our results suggest that in at least a subpopulation of amygdala neurons, this might not be the case. Indeed, it is likely that in this spared population, the remnant *Shh *expression domain in the embryonic caudal telencephalon in *Nkx2-1*-mutant mice is sufficient to directly specify these inhibitory neurons directly.

### Fate-mapping of embryonic *Gli1*^*+ *^cells reveal the precocious generation of inhibitory neural diversity in both subdivisions of the posterior MeA compared to other forebrain structures

*Shh *signaling is mediated through the *Gli *family of transcription factors, which are homologous to the *Drosophila *zinc finger transcription factor cubitus interruptus, which mediates all Hedgehog signaling in the fly [62]. Of the three mammalian *Gli *family members, *Gli1 *and *Gli2 *primarily act as activators whereas *Gli3 *functions as a repressor [63-65]. Analysis of loss of function mutant mice has shown that *Shh *signaling is required for the initial transcriptional activation of endogenous *Gli1*, but not *Gli2*, in the forebrain [24]. Here, by administering a single tamoxifen dose at E9.5, we were able to label *Gli1*^*+ *^*Shh*-responder cells born from approximately E10.5 in the *Gli1*^*CreER(T2) *^line, a readout of *Shh *activity [21]. From this analysis, we show that cells that respond to *Shh *signaling also contribute to inhibitory neural diversity in the posterior MeA. Co-expression was observed with all inhibitory markers used in this study: Calbindin, nNOS, FoxP2 and SST. Furthermore, numerous LacZ^+ ^recombined cells in the MePD and MePV in *Gli1*^*CreER(T2)*^*; Tau*^*mGFP *^(TM E9.5) brains lend credence to prior observations that indicate that neurons of the MeA are born earlier in development than those destined for other amygdala nuclei, such as those of the basolateral complex [3,4,7,8].

## Conclusions

In this study, we have shown that *Nkx2-1*- and *Shh*-lineage cells preferentially fate-map to the dorsal and ventral subdivisions of the posterior MeA, with differential contributions to both local and projection inhibitory neurons. Previous tracing studies have identified that MePD efferent neurons project to three interconnected nuclei that are involved in reproductive behaviors: the medial preoptic nucleus, the ventrolateral part of the ventromedial hypothalamic nucleus and the ventral premamillary nucleus. In contrast, projections from the MePV involved in defensive behaviors terminate in the anterior hypothalamic nucleus and the dorsomedial portion of the ventromedial hypothalamic nucleus [12,66]. In a behavioral context, the reproductive and defensive actions are closely interrelated, and upon the appearance of threatening behaviors, a 'gate-control' mechanism ensures the rapid shut-down of reproductive behaviors to aid survival. The use of different *Lhx *transcription factors that delineate these projections to their hypothalamic targets may potentially serve as a neural substrate to integrate conflicting reproductive and defensive behavioral cues [12]. Here, we show the inhibitory neural diversity of these nuclei is generated from *Shh*-expressing and *Shh*-responsive cells, implicating the *Shh-*pathway component of MeA development. Therefore, the data from the current study provide novel insights into the gene network complexity and genetic mechanisms involved in the development of the MeA.

## Materials and methods

### Animal use

Mouse lines used in this study were: Swiss-Webster (SW; Taconic Farms, Albany, NY, USA), *Nkx2-1 *mutant [18], *Shh*^*Cre *^[22], *ROSA-YFP *[67], *Tau*^*mGFP *^[28], *Gli1*^*CreER(T2) *^[21] and *Nkx2-1-Cre *[5]. Mice were maintained according to protocols approved by the Animal Welfare and use committee at Children's National Medical Center, Washington DC, National Institutes of Health, Bethesda, MD, and NYU School of Medicine, NY. *Nkx2-1 *mutant mice were maintained on a SW background. The *Nkx2-1-Cre *and *Shh*^*Cre *^lines were maintained on a C57Bl/6 background, the *Gli1*^*CreER(T2) *^mice were kept on a SW background and the reporter mice on C57Bl/6 × SW mixed backgrounds.

### Animal crosses and genotyping

For staging of the embryos, midday of the day of vaginal plug detection was considered as E0.5. The day of birth was considered P0. To avoid potential sexual dimorphic differences in the MeA, only males were used for postnatal analyses. Mice were transcardially perfused with 4% paraformaldehyde (PFA) and brains were post-fixed in 4% PFA for at least 6 hours to overnight. Pregnant females from *Gli1*^*CreERT2*^; *Tau*^*mGFP *^crosses were administered a single dose (100 mg/kg in corn oil) of tamoxifen (Sigma, St Louis, MO, USA) by oral gavage at E9.5, and embryos collected at E12.5 or pups were perfused at P22. For early embryonic analysis (E10.5 to E13.5) pregnant dams were euthanized by CO_2 _inhalation and the embryos were harvested by Caesarian section and whole heads were fixed for 2 hours (for X-gal staining) or overnight in 4% PFA at 4°C. Pregnant dams of E18.5 litters were anaesthetized with Nembutal and the embryos were transcardially perfused. The isolated brains were post-fixed overnight at 4°C. Genomic DNA for genotyping was isolated by phenol:chloroform extraction. Mice for fate-mapping and electrophysiological analyses were identified by PCR for Cre [68] and yellow fluorescent protein (YFP) [69] for transgenic and reporter lines, respectively. *Nkx2-1 *mutant embryos were identified by PCR using a GC rich kit (Roche, Indianapolis, IN, USA) and previously described primers [18,23], and morphologically by the absence of lungs [37].

### Tissue preparation and histology

After rinsing in PBS, postnatal brains were embedded in 4% agarose and sectioned coronally at a thickness of 50 μm using a Vibroslicer (Leica VT1000S, Leica, Nussloch, Germany). Embryonic brains were cryoprotected by graded sucrose immersion (10%, 20% then 30% overnight) and embedded in Tissue-Tek OCT Compound (Sakura Finetek USA Inc., Torrance, CA, USA). Coronal sections at a thickness of 20 (E10.5 to E13.5) or 30 μm (E18.5) were collected on glass slides, air-dried and stored at -20°C.

### Non-radioactive dioxygenin-labeled RNA *in situ *hybridization

mRNA *in situ *hybridization was carried out as described previously [68] with the exception that the reaction product was visualized by NBT/BCIP (Roche) diluted in AP buffer (10 μl/100 mM NaCl, 100 mM Tris pH 9.5, 50 mM MgCl_2_). The following cDNA probes were used in this study: *Cck*, *Foxg1*, *GAD67*, *Gli1*, *Lhx6*, *Lhx9*, *Nkx2-1*, *Shh *and *Sst*.

### Immunofluorescence

Free-floating adult vibratome sections were rinsed in PBS prior to blocking in 10% normal serum (NS) in PBS with Triton 0.03% (PBST; Sigma) for 1 hour at room temperature (RT), then overnight incubation in the primary antibodies diluted in PBST with 1% NS at RT for free-floating or 4°C for mounted tissue. The sections underwent three 10-minute rinses in PBS and were incubated in the appropriate donkey anti-Cy3, Cy5 (1:200; Jackson Immunoresearch, West Grove, PA, USA) or donkey anti-Alexa 488 (Invitrogen, Carlsbad, CA, USA) secondary antibodies, diluted in PBST with 1% normal donkey serum (NDS) for 2 hours at RT. The sections were rinsed twice then incubated in DAPI (1:1,000 in PBS; Sigma) for 10 minutes. The sections were mounted on Superfrost slides and coverslipped using Gel Mount aqueous mounting media (Sigma). The primary antibodies used in distinct combinations were: goat anti-β-gal (1:500; Biogen, Cambridge, MA, USA), goat anti-FoxP2 (1:500; Santa Cruz Biotechnology Inc., Santa Cruz, CA, USA), goat anti-GFP (1:1,000; Novus Biologicals, Littleton, CO, USA), rabbit anti-β-gal (1:1,000; ICN Pharmaceuticals Inc, Costa Mesa, CA, USA), rabbit anti-Calbindin (1:1,000; Calbiochem, La Jolla, CA, USA), rabbit anti-nNOS (1:1,000; Sigma), rat anti-GFP (1:1,000; Nacalai USA, San Diego, CA, USA) and rat anti-SST (1:250; Millipore, Billerica, MA, USA).

### Permanent immunohistochemistry

Free-floating adult vibratome sections and E18.5 cryostat sections were rinsed in PBS and incubated in PBS:methanol:30% H_2_O_2 _(Sigma) in an 8:1:1 ratio for 20 minutes at RT to quench endogenous peroxidases. The sections underwent further rinses in PBS prior to blocking of non-specific binding sites using 10% NS in PBST. The primary antibodies were incubated overnight at RT. Further rinses in PBS preceded and followed incubations for 1 hour at RT in biotinylated antibodies (1:500; Vector Labs, Burlingame, CA, USA) then extravidin-peroxidase (1:2,000; Sigma), all diluted in 1% NS in PBST. The reaction product was visualized using a DAB kit (Vector Labs) according to the manufacturers' instructions. The reaction was stopped in Tris-buffered saline pH7.5 and the sections were mounted and/or coverslipped using permanent mounting media (Sigma). The primary antibodies used were as follows: goat anti-FoxP2 (1:1,000), goat anti-GFP (1:1,000), mouse anti-Parvalbumin (1:1,000; Sigma), rabbit anti-Calbindin (1:2,000), rabbit anti-nNOS (1:3,000), rabbit anti-Tbr1 (1:1,000; kind gift of R Hevner), rabbit anti-Neuropeptide Y (1:1,000; Immunostar, Hudson, WI, USA), rabbit anti-GAD65/67 (1:1,000; Millipore), and rat anti-SST (1:250).

### Electrophysiology

Electrophysiological analysis of *Shh*-lineage cells in the posterior MeA was performed on male mice at P17 to P21 from *Shh*^*Cre*^*; ROSA26-YFP *crosses. Mice were anesthetized with isoflurane, decapitated, and their brains were removed. Brain hemispheres were rapidly dissected on ice and cut into 300-μm thick coronal sections on a Leica VT100S vibratome in an ice-cold oxygenated solution consisting of (in mM) 87 NaCl, 2.5 KCl, 1.25 NaH_2_PO_4_, 7 MgCl_2_, 0.5 CaCl_2_, 25 NaHCO_3_, 25 glucose, 75 sucrose (347 mOsmol) at pH 7.4. Slices were stored in the same solution at 35°C for 30 minutes, then transferred into artificial cerebrospinal fluid of the following composition (in mM): 124 NaCl, 3 KCl, 2.5 CaCl_2_, 1.3 MgSO_4_, 26 NaHCO_3_, 1.25 NaHPO_4_, 15 glucose; saturated with 95% O_2_/5% CO_2 _at room temperature (20 to 25°C).

Slices were transferred to the recording chamber and perfused with artificial cerebrospinal fluid at a rate of 1 to 2 ml/s. Cells were visualized using an upright Olympus BX51W microscope equipped with infrared and fluorescent illumination, Normasky optics, and infrared camera (all from Olympus). Cells were only chosen based on their YFP fluorescence and apparent viability (clear membrane, nucleus not visible) without bias in terms of location within the MePD or MePV, cell size or morphology. Patch electrodes had resistances between 3 and 6 MΩ when filled with the intracellular solution of the following composition (in mM): 130 K-gluconate, 10 NaCl, 2 Mg-ATP, 0.3 Na-GTP, 10 HEPES, 0.6 EGTA, biocytin 5 mg/ml, solution adjusted to pH 7.2, 275 mOsm (junction potential = 13 mV). Whole-cell recordings were obtained using a Multiclamp 700B (Molecular Devices, Sunnyvale, CA, USA) and recordings were monitored via a PC running pClamp 9.2 (Molecular Devices). After recording membrane potential, capacitance and resistance values, cells were categorized on the basis of their response to depolarizing and hyperpolarizing current pulses. Off-line analysis was performed using Clampfit 9.2 (Molecular Devices) and Mini Analysis (Synaptosoft Inc., Fort Lee, NJ, USA). In all experiments, data were filtered at 10 kHz during capacitance compensation and 5 kHz during subsequent data recording. The traces were digitized at 10 kHz. All voltage measurements and steps were corrected for a junction potential offset.

### Data analysis

Analysis of *in situ *hybridization experiments was performed using bright-field microscopy (Olympus BX51, Olympus, Center Valley, PA, USA) and high-resolution digital images were captured under a 4× objective using an Olympus D570 camera. For fluorescence, digital photographs were obtained from epifluorescence microscopy (Olympus BX61) and selected for further analysis using a Zeiss LSM 510 META confocal microscope (Thornwood, NY, USA). For confocal analysis, each fluorophore was scanned sequentially and confocal images are presented as individual optical sections. Figures were prepared using Adobe Photoshop CS and Adobe Illustrator CS software (Adobe Systems, San Jose, CA, USA). Adjustments to contrast were applied across each image as a whole and equally to control and mutant brains. For anatomical considerations we included the embryonic expression domain terminology of Flames and colleagues [19]. For the adult anatomy we used the atlas of Franklin and Paxinos [70]. General anatomical consultations were made from prior publications on the posterior MeA [2,4,33,41]. All *n *values for immunohistochemistry, *in situ *hybridization and electrophysiology are detailed in each corresponding figure legend.

### Cell quantification

To assess the co-expression of immunohistochemical markers in recombined cells from fate-mapping analysis in adults from the three Cre lines, we performed cell counts in the MePD and MePV from two rostro-caudal levels of the posterior MeA, corresponding to Bregma levels -1.46 mm and -1.82 mm. Cells were counted in a 400 μm × 400 μm boxed area through 10 adjacent sections (1-μm steps) of each Z-stack using the LSM510 software. For the fate-mapping analysis we used the *Tau*^*mGFP *^reporter mouse, which was designed with a nuclear localization signal for LacZ [28]. Therefore, we used an anti-β-gal antibody for nuclear staining of recombined cells, which, along with DAPI counterstaining, facilitated quantification of cells expressing other makers that were also primarily nuclear. The numerical data from the fate-mapping analysis are presented as the average percentage and standard deviation of recombined β-gal^+ ^cells that co-expressed the second marker. Statistical significance was determined using an unpaired *t*-test (alpha value was set at 0.05) and was calculated using online GraphPad software (GraphPad Software Inc., La Jolla, CA, USA).

## Abbreviations

β-gal: β-galactosidase; E: embryonic day; LGE: lateral ganglionic eminence; MeA: medial amygdala; MePD: medial posterodorsal nucleus; MePV: medial posteroventral nucleus; MGE: medial ganglionic eminence; nNOS: neuronal nitric oxide synthase; NS: normal serum; P: postnatal day; PBS: phosphate-buffered saline; PBST: PBS with Triton 0.03%; PFA: paraformaldehyde; pPOA: progenitor domain of the preoptic area; POA: preoptic area; RT: room temperature; *Shh*: *Sonic hedgehog*; SST: Somatostatin; SW: Swiss-Webster; TM: tamoxifen; YFP: yellow fluorescent protein.

## Competing interests

The authors declare that they have no competing interests.

## Authors' contributions

RSEC and JGC devised the study. RSEC performed all of the experiments with the exception of the electrophysiology, and was the primary contributor to the design, data analysis and figure preparation. J-MM performed the electrophysiology experiments and analysis. KM provided further technical assistance, and along with LH, VHS and RPM provided preliminary data. LH and RPM generated the *Gli1*^*CreER(T2) *^and *Nkx2-1-Cre *tissue, respectively. VG, SA and GF provided reagents and gave intellectual input to the study. RSEC wrote the majority of the manuscript with minor contributions from JM-M (electrophysiology methods and results) and JGC. VG, SA and GF further edited and all authors approved the manuscript.

## Supplementary Material

Additional file 1 **Expression patterns of excitatory and inhibitory neuronal markers in the MePD and MePV**. **(A-H) **Permanent immunohistochemistry in P22 wild-type brains (*n *= 3) showing the expression of known excitatory and inhibitory neuronal markers in the MePD (A,C,E,G) and MePV (B,D,F,H) of the posterior MeA. (A,B) Tbr1 expression (arrows) is largely devoid in these nuclei, which have primarily a GABAergic neuronal projection output, as indicated by intense GAD65/67 expression (C,D, arrows). (E,H) Interestingly, classic inhibitory markers such as Parvalbumin (E,F, arrows) and Neuropeptide Y (NPY; G,H, arrows) are also sparsely observed in the MePD and MePD. Abbreviations: I, intercalated nuclei of the amygdala; opt, optic tract; slME, superficial layer of the medial nucleus. Scale bar: 200 μm (A-H). Click here for file

Additional file 2 ***Shh*^*Cre *^recombination in the *RYFP *reporter mouse line**. **(A,B) **Permanent immunohistochemistry for GFP in *Shh*^*Cre*^*; RYFP *brains (*n *= 2) shows that this reporter mouse shows the same distribution of *Shh*-lineage cells in the adult MePD (A, arrows) and MePV (B, arrows) as the *Tau*^*mGFP *^reporter line used in this study. Abbreviations: opt, optic tract; slME, superficial layer of the medial nucleus. Scale bar: 200 μm (A-H). Click here for file

Additional file 3 **Intrinsic electrophysiological properties of *Shh*-lineage cells in the posterior medial amygdala**. Intrinsic electrophysiological properties of *Shh*-lineage cells in the posterior medial amygdala. Click here for file
