# Supplementary figures and images for: Sonic hedgehog expressing and responding cells generate neuronal diversity in the medial amygdala
Source: Neural Dev. 2010 May 27;5:14. doi: 10.1186/1749-8104-5-14 (PMC2892491; doi:10.1186/1749-8104-5-14)

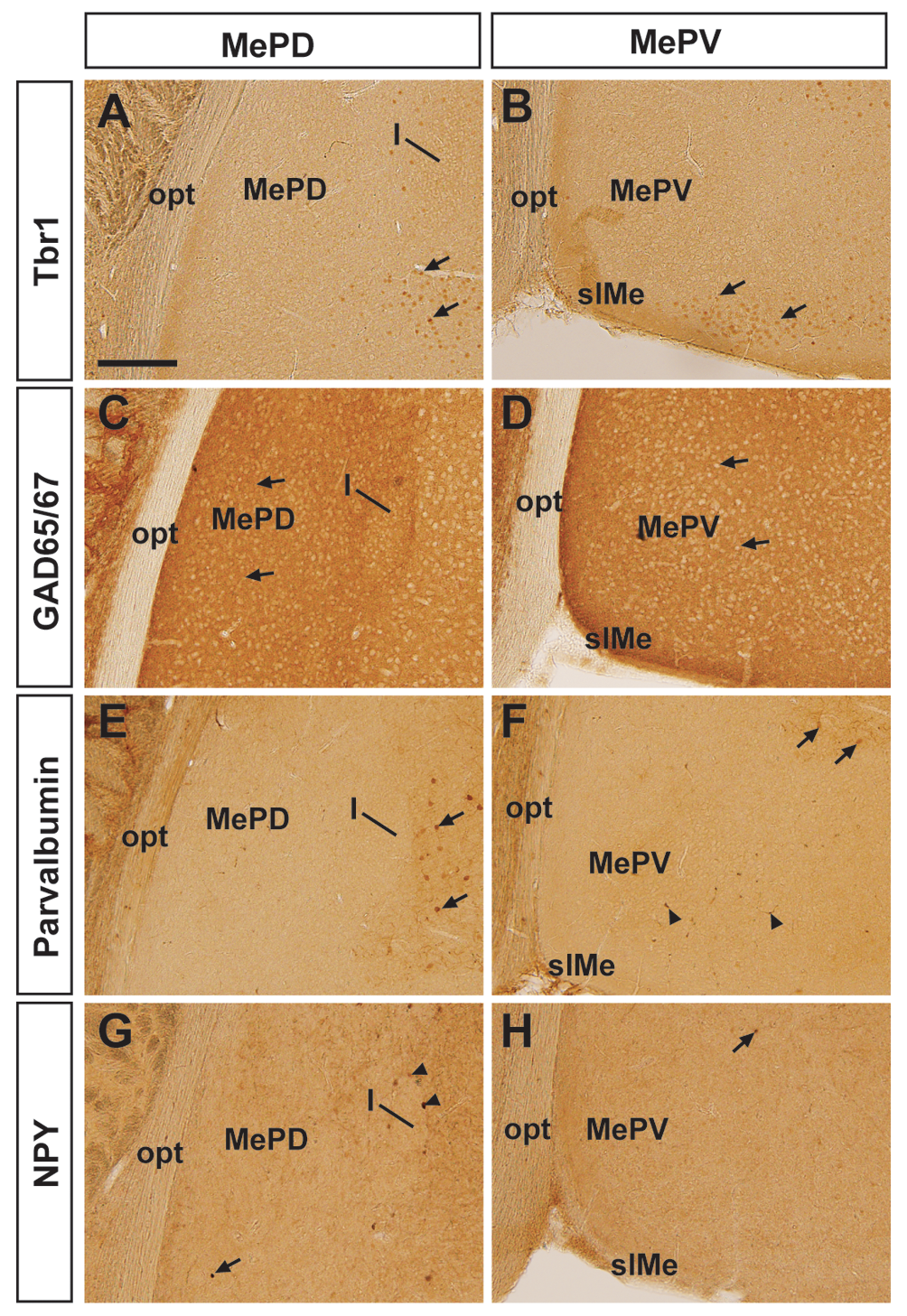

Supplement: Additional file 1 — Expression patterns of excitatory and inhibitory neuronal markers in the MePD and MePV. (A-H) Permanent immunohistochemistry in P22 wild-type brains (n = 3) showing the expression of known excitatory and inhibitory neuronal markers in the MePD (A,C,E,G) and MePV (B,D,F,H) of the posterior MeA. (A,B) Tbr1 expression (arrows) is largely devoid in these nuclei, which have primarily a GABAergic neuronal projection output, as indicated by intense GAD65/67 expression (C,D, arrows). (E,H) Interestingly, classic inhibitory markers such as Parvalbumin (E,F, arrows) and Neuropeptide Y (NPY; G,H, arrows) are also sparsely observed in the MePD and MePD. Abbreviations: I, intercalated nuclei of the amygdala; opt, optic tract; slME, superficial layer of the medial nucleus. Scale bar: 200 μm (A-H). [file 1749-8104-5-14-S1.TIFF]

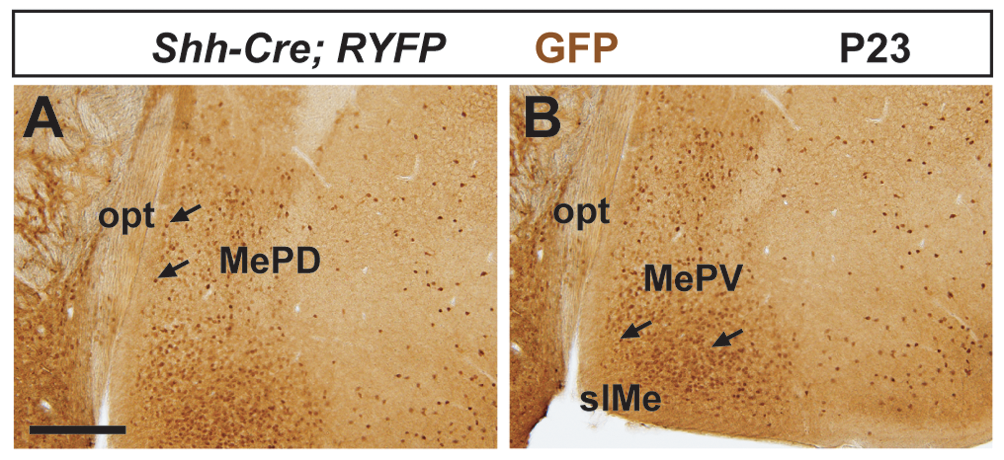

Supplement: Additional file 2 — ShhCre recombination in the RYFP reporter mouse line. (A,B) Permanent immunohistochemistry for GFP in ShhCre; RYFP brains (n = 2) shows that this reporter mouse shows the same distribution of Shh-lineage cells in the adult MePD (A, arrows) and MePV (B, arrows) as the TaumGFP reporter line used in this study. Abbreviations: opt, optic tract; slME, superficial layer of the medial nucleus. Scale bar: 200 μm (A-H). [file 1749-8104-5-14-S2.TIFF]
