# Supplementary material for: Sonic hedgehog expressing and responding cells generate neuronal diversity in the medial amygdala
Source: Neural Dev. 2010 May 27;5:14. doi: 10.1186/1749-8104-5-14 (PMC2892491; doi:10.1186/1749-8104-5-14)
Supplement: Additional file 3 — Intrinsic electrophysiological properties of Shh-lineage cells in the posterior medial amygdala. Intrinsic electrophysiological properties of Shh-lineage cells in the posterior medial amygdala. [file 1749-8104-5-14-S3.DOC]

**Additional file 3 - Intrinsic electrophysiological properties of *Shh***-lineage cells in the posterior medial amygdala

|  | **Class I**  (Nos^+^/FoxP2^-^) | Class II (Nos^+^/FoxP2^+^) | **Class III**  (Nos^-^/FoxP2^-^) |
| --- | --- | --- | --- |
| **Membrane resistance (MΩ)** | 501 ± 213 | 474 ± 153 | 410 ± 74 |
| **Membrane capacitance (pF)** | 53 ± 7 | 60 ± 17 | 51 ± 18 |
| **Membrane potential (mV)** | -57 ± 3 | -58 ± 5 | -54 ± 3 |
| **Peak amplitude (mV)** | 45 ± 6 | 38 ± 6 | 39 ± 4 |
| **Half-spike duration for 1^st^**  **Spike (ms)** | 1.77 ± 0.36 | 1.9 ± 0.29 | 1.94 ± 0.23 |
| **Maximum frequency (Hz)** | 24 ± 5 | 1.5 ± 0.7 | 11 ± 5 |
| **Accommodation ratio** | 0.46 ± 0.08 | Not Applicable | Not Applicable |
| **Sag (mV)** | 5.5 ± 2 | 0.7 ± 0.5 | 1.5 ± 0.8 |
| **I_T_-like current (mV)** | 1.5 ± 1 | 18 ± 6 | 10 ± 5 |
| **AHP amplitude (mV)** | -11.2 ± 2.5 | -12.3 ± 1.9 | -13.2 ± 1.5 |
| **AHP decay half-time (ms)** | 50.7 ± 14.7 | 10.6 ± 5 | 15.4 ± 4.2 |
| **Number of neurons recorded**  **(Total = 28)** | *n* = 9 | *n* = 8 | *n* = 11 |
